# CEACAM7 Expression and DNA Methylation: Prognostic Biomarkers for Lung Adenocarcinoma in African Americans

**DOI:** 10.1016/j.jtho.2025.08.015

**Published:** 2025-08-22

**Authors:** Alicia Hulbert, Richies Tiv, Brent Cao, Daniel Cesario, Apurva Mallisetty, Eric Gauchat, Ajay Rana, Frank Weinberg, Ludmila Danilova, Ignacio Jusue-Torres

**Affiliations:** a Department of Surgery, University of Illinois at Chicago, College of Medicine, Chicago, Illinois; b University of Illinois Hospital and Health Sciences System Cancer Center, University of Illinois at Chicago, Chicago, Illinois; c University of Wisconsin School of Medicine and Public Health, Madison, Wisconsin; d Division of Hematology and Oncology, Department of Medicine, College of Medicine, University of Illinois of Chicago, Chicago, Illinois; e Department of Oncology, The Sidney Kimmel Comprehensive Cancer Center Johns Hopkins University School of Medicine, Baltimore, Maryland; f Department of Neurosurgery, Mayo Clinic, Rochester, Minnesota

**Keywords:** Lung cancer, Epigenetics, DNA methylation, Survival, Disparities

## Abstract

**Introduction:**

The goals of this study were to identify lung cancer biomarkers specific to African Americans (AA) by conducting a genome-wide analysis and to understand the role of these biomarkers in overall survival.

**Material and Methods:**

All cancers studied by The Cancer Genome Atlas were queried with the TCGAbiolinks R package (RRID:SCR_017683) for mRNA expression, DNA methylation, and clinical data. Differential expression analysis was performed comparing mRNA expression between AA and Whites. Survival duration differences were studied using the 2-tailed log-rank test. Association with survival was quantified using hazard ratios with 95% confidence intervals with univariate and multivariate Cox proportional hazard models. Spearman’s rank correlation analysis was used to identify CpG sites correlated with mRNA expression.

**Results:**

CEACAM7 was the only gene overexpressed among AA compared with Whites with lung adenocarcinoma (LUAD), and its RNA expression was significantly associated with cancer stage, overall survival duration, and mortality risk in AA. DNA methylation on CpG probes cg22895231, cg25465322, cg01656853, cg17659920, and cg07940585 was negatively correlated with CEACAM7 mRNA expression, directly associated with survival duration, and inversely associated with mortality risk in AA with LUAD. Median survival was 37.29 months versus not reached for the high versus low CEACAM7 expression groups. The 1-, 2-, and 5-year survival rates for the high versus low CEACAM7 mRNA expression were 80% versus 100%, 50% versus 100%, and 20% versus 86%, respectively.

**Conclusion:**

CEACAM7 mRNA expression and its DNA methylation may be associated with overall survival duration and mortality risk among AA with LUAD but not in Whites.

## Introduction

In the past several decades, lung cancer has been the leading cause of death with an estimated 1.23 million deaths and an estimated 1.57 million cases globally.^1,2^ As such, there is significant demand for insight into lung cancer genomics. The Cancer Genome Atlas (TCGA) was established by the National Cancer Institute (NCI) and the National Human Genome Research Institute to provide a coordinated effort to characterize primary cancers, including—of course—lung cancer. Since the public release of TCGA data sets, hundreds of studies have been published, including discoveries of a multitude of novel biomarkers in lung cancer.^3^ Biomarkers, such as EGFR, have played a pivotal role in treating lung cancer with targeted therapy. However, approximately 10% to 20% of patients with lung cancer harbor EGFR mutations.^4–9^ Furthermore, racial health disparities in lung cancer continue to be a clinical challenge. African Americans (AA) carry a disproportionate share of the lung cancer burden, with higher incidence, later-stage diagnosis, and poorer 5-year overall survival rates when compared with Whites.^10–14^ A recent study performing genomic analysis by parallel sequencing of 504 cancer genes in 509 lung cancer tumor specimens did not reveal significant differences in the mutational profiles between AA and Whites, suggesting that epigenetic, rather than genetic, differences may be underlying the racial disparities.^15^ Therefore, given that a large proportion of patients with lung cancer do not harbor any of the cancer driver genes, racial disparities in lung cancer are not currently well explained by differences in genetic mutations. As such, the goal of our study is to identify lung cancer biomarkers specific to AA by conducting a genome-wide analysis, understand the role these biomarkers play in overall survival, and discover how epigenetic modulations can have an impact in the cancer process.

## Material and Methods

### Clinical Data

We analyzed the clinical data of 522 patients with lung adenocarcinoma (LUAD) and 504 patients with lung squamous cell carcinoma (LUSC) from the TCGA through the TCGAbiolinks R package (RRID: SCR_017683) (2.30.4).^16–18^ Collected variables included age, sex, race, pack*year smoked, years smoked, prior malignancy, histology, and American Joint Committee on Cancer (AJCC) cancer stage.^19^ Baseline demographic characteristics of the groups are presented as median (interquartile range) for continuous variables or n (%) for categorical variables and were compared using the Wilcoxon ranked sum test for continuous variables and Fisher’s exact test for categorical variables. Statistical significance was defined as a *p* value less than 0.05. Statistical analysis was performed using R version 4.1.1 (RRID:SCR_001905).^20^ The reporting of this study conforms to the Strengthening the Reporting of Observational Studies in Epidemiology statement and the Reporting Recommendations for Tumor Marker Prognostic Studies (REMARK).^21,22^

### Differential mRNA Expression Analysis

We accessed mRNA transcripts for 32 cancer types available on the TCGA repository (adrenocortical carcinoma [ACC], bladder urothelial carcinoma [BLCA], breast invasive carcinoma [BRCA], cervical squamous cell carcinoma and endocervical adenocarcinoma [CESC], cholangiocarcinoma [CHOL], colon adenocarcinoma [COAD], lymphoid neoplasm diffuse large B-cell lymphoma [DLBC], esophageal carcinoma [ESCA], glioblastoma multiforme [GBM], head and neck squamous cell carcinoma [HNSC], kidney chromophobe [KICH], kidney renal clear cell carcinoma [KIRC], kidney renal papillary cell carcinoma [KIRP], lower grade glioma [LGG], liver hepatocellular carcinoma [LIHC], LUAD, LUSC, mesothelioma [MESO], ovarian serous cystadenocarcinoma [OV], pancreatic adenocarcinoma [PAAD], pheochromocytoma and paraganglioma [PCPG], prostate adenocarcinoma [PRAD], rectum adenocarcinoma [READ], sarcoma [SARC], skin cutaneous melanoma [SKCM], stomach adenocarcinoma [STAD], testicular germ cell tumors [TGCT], thyroid carcinoma [THCA], thymoma [THYM], uterine corpus endometrial carcinoma [UCEC], uterine carcinosarcoma [UCS], and uveal melanoma [UVM]) sequenced on the Illumina HiSeq platform using the TCGAbiolinks R package (2.30.4)^16–18^ and performed a differential expression (DE) analysis using the DESeq package (1.42.1) (RRID:SCR_000154). The mRNA transcripts were processed through an Array Array Intensity Correlation (AAIC) using a cutoff of 0.6. These transcripts were normalized through the EDASeq package based on gcContent (RRID:SCR_006751).^23^ Differential expression analysis was only performed for those repositories where there were 2 or more patients in both the Whites and AA cohorts. We performed DE analysis comparing RNA expression in AA versus Whites. CEACAM7 mRNA expression group allocation was based on DE analysis, comparing low versus high expression in AA only, by dichotomizing gene expression values based on the median without randomization. Genes with a false discovery rate (FDR)–corrected *p* value less than 10^−5^ and an absolute log_2_FC more than 3 were considered significant.

### DNA Methylation Data

The DNA methylation data for 473 LUAD and 370 LUSC primary tumor samples were previously generated by TCGA using the Illumina Human Methylation 450 platform. We accessed DNA methylation beta values through the TCGAbiolinks R package (2.30.4).^16–18^ The samples were dichotomized into low and high DNA methylation based on the median beta value of the corresponding CpG site.

### Survival Analysis

Two-tailed log-rank tests were used to analyze overall survival differences comparing low and high mRNA expression with low and high DNA methylation in AA and Whites patients. Furthermore, univariate and multivariate Cox proportional hazard models were used to obtain hazard ratios (HRs) (95% confidence intervals [CIs]) to study the association with mortality risk. Statistical significance was defined as a *p* value less than 0.05. Statistical analysis was performed using version 4.1.1 of the R language.^20^ These survival analysis methods were extended to a pan-cancer analysis comparing low and high mRNA expression with low and high DNA methylation in AA and Whites patients for ACC, BLCA, BRCA, CESC, CHOL, COAD, DLBC, ESCA, GBM, HNSC, KICH, KIRC, KIRP, LGG, LIHC, LUAD, LUSC, MESO, OV, PAAD, PCPG, PRAD, READ, SARC, SKCM, STAD, TGCT, THCA, THYM, UCEC, UCS, and UVM. The same methodology was used to perform co-expression survival analyses of CEACAM7 and IL1A on all 32 cancer types in which there were at least two or more patients in their respective repositories.

### DNA Methylation Probe Identification for CEACAM7

Spearman’s rank correlation analysis was used to identify CpG sites on chr19 correlated with the mRNA expression of CEACAM7 (r < −0.35, *p* value ≤ 0.01). Univariate Cox proportional hazard models were used to identify statistically significant CpG sites associated with mortality risk (*p* value ≤ 0.01).

### CEACAM7 Normal Tissue RNA Expression

CEACAM7 RNA expression data from more than 50 different normal primary tissues were obtained from the Genotype-Tissue Expression (GTEx) Project Portal dbGaP accession phs000424.v8.p2 GSE2361.^24–27^

## Results

### Patient Clinical Characteristics

**Figure 1.**
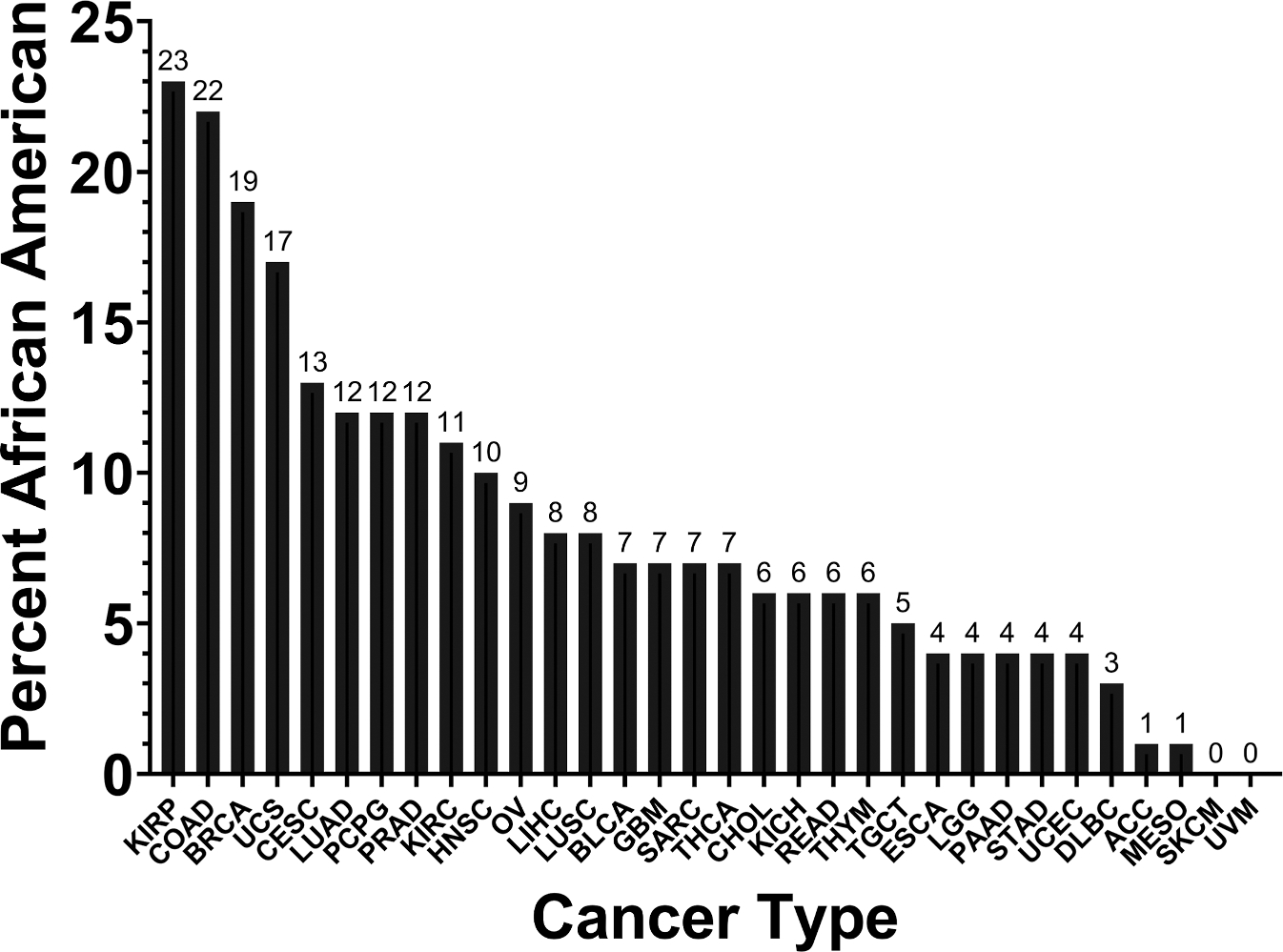
Proportion of African Americans by cancer type in TCGA revealing 12% and 8% of African American among LUAD and LUSC, respectively. LUAD, lung adenocarcinoma; LUSC, lung squamous cell carcinoma; TCGA, The Cancer Genome Atlas.

TCGA collected data for 522 patients with LUAD and 504 patients with LUSC. The median age at diagnosis was 67 (59–73) years in LUAD and 69 (62–74) years in LUSC. For LUAD, there were 54% females and 75% Whites with the following breakdown of other race categories: unreported (13%), AA (12%), Asian (2%), and American Indian or Alaska Native (<1%). For LUSC, there were 26% females and 70% Whites with the following breakdown of other race categories: unreported (22%), AA (8%), and Asian (2%). The percentage of AA across all cancer types in TCGA ranged from 0% in SKCM and UVM to 23% in KIRP, and it was 12% and 8% for LUAD and LUSC, respectively (Fig. 1). The AJCC pathologic stages for LUAD were 53% (stage I), 24% (stage II), 16% (stage III), and 5% (stage IV) and for LUSC were 49% (stage I), 32% (stage II), 17% (stage III), and 1% (stage IV), respectively.

### Differentially Expressed Genes in African Americans

**Figure 2.**
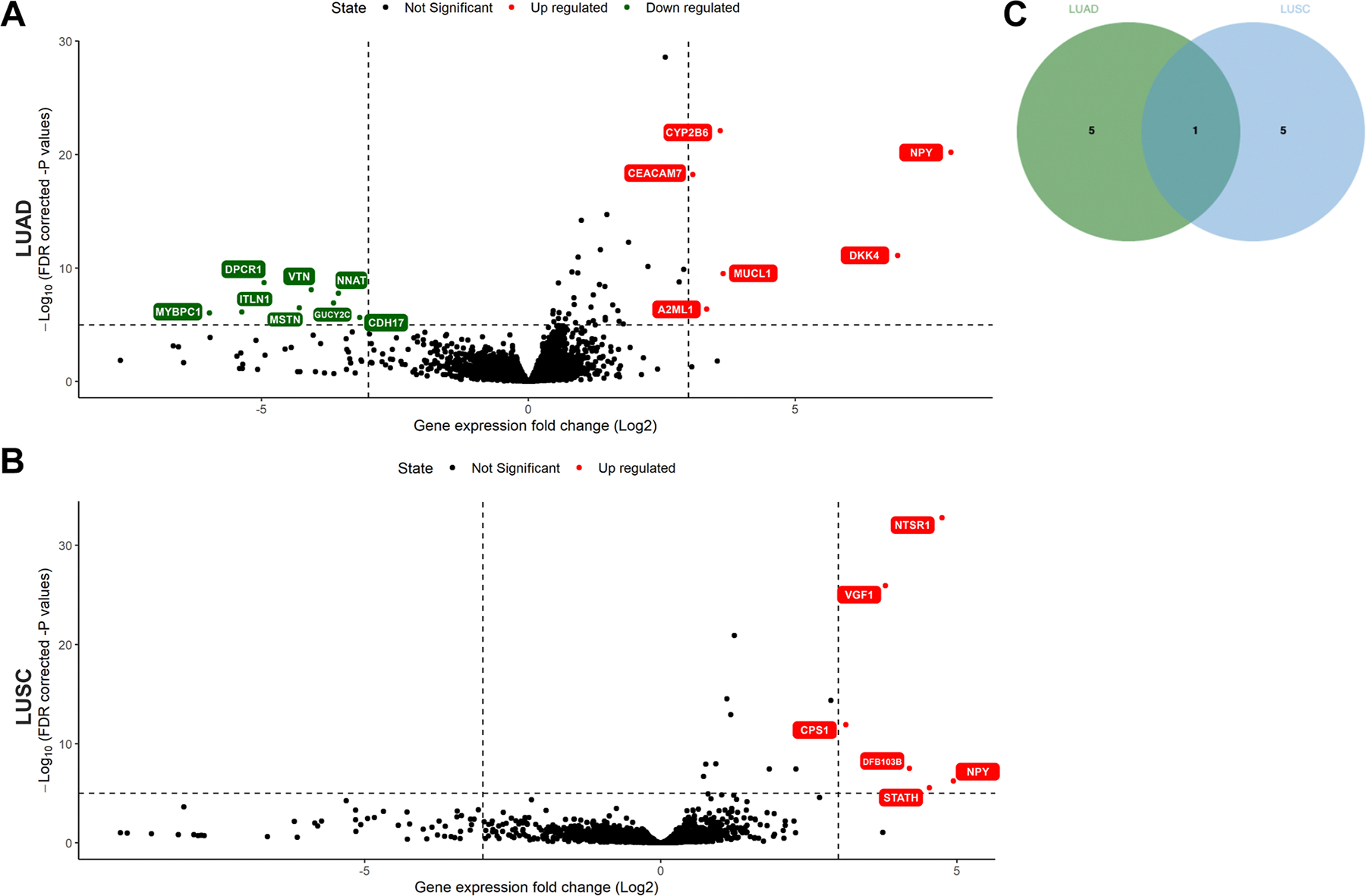
CEACAM7 is overexpressed in African Americans with LUAD when compared with Whites. (*A*) Volcano plots for down- and up-regulated genes in African Americans compared with Whites for LUAD and (*B*) LUSC (FDR cutoff = 10^−5^, log_2_FC = 3). (*C*) Venn diagram presenting the shared up-regulated genes in LUAD and LUSC. FDR, false discovery rate; LUAD, lung adenocarcinoma; LUSC, lung squamous cell carcinoma.

We performed a differential expression analysis on AA versus Whites in LUAD and AA versus Whites in LUSC (Fig. 2A and B). Among the patients with LUAD, we identified six up-regulated genes (CEACAM7, A2ML1, CYP2B6, DKK4, MUCL1, and NPY) in AA. Among the patients with LUSC, we also identified six up-regulated genes (CPS1, DEFB103B, NPY, NTSR1, STATH, and VGF) in AA. In addition, we identified eight down-regulated genes (CDH17, DPCR1, GUCY2C, ITLN1, MSTN, MYBPC1, NNAT, and VTN) in AA with LUAD (Fig. 2A) and no down-regulated genes in AA with LUSC (Fig. 2B). Of the up-regulated genes in AA, there was just one gene that was up-regulated in both LUAD and LUSC (NPY) (Fig. 2C).

### CEACAM7 RNA Expression by Stage

**Figure 3.**
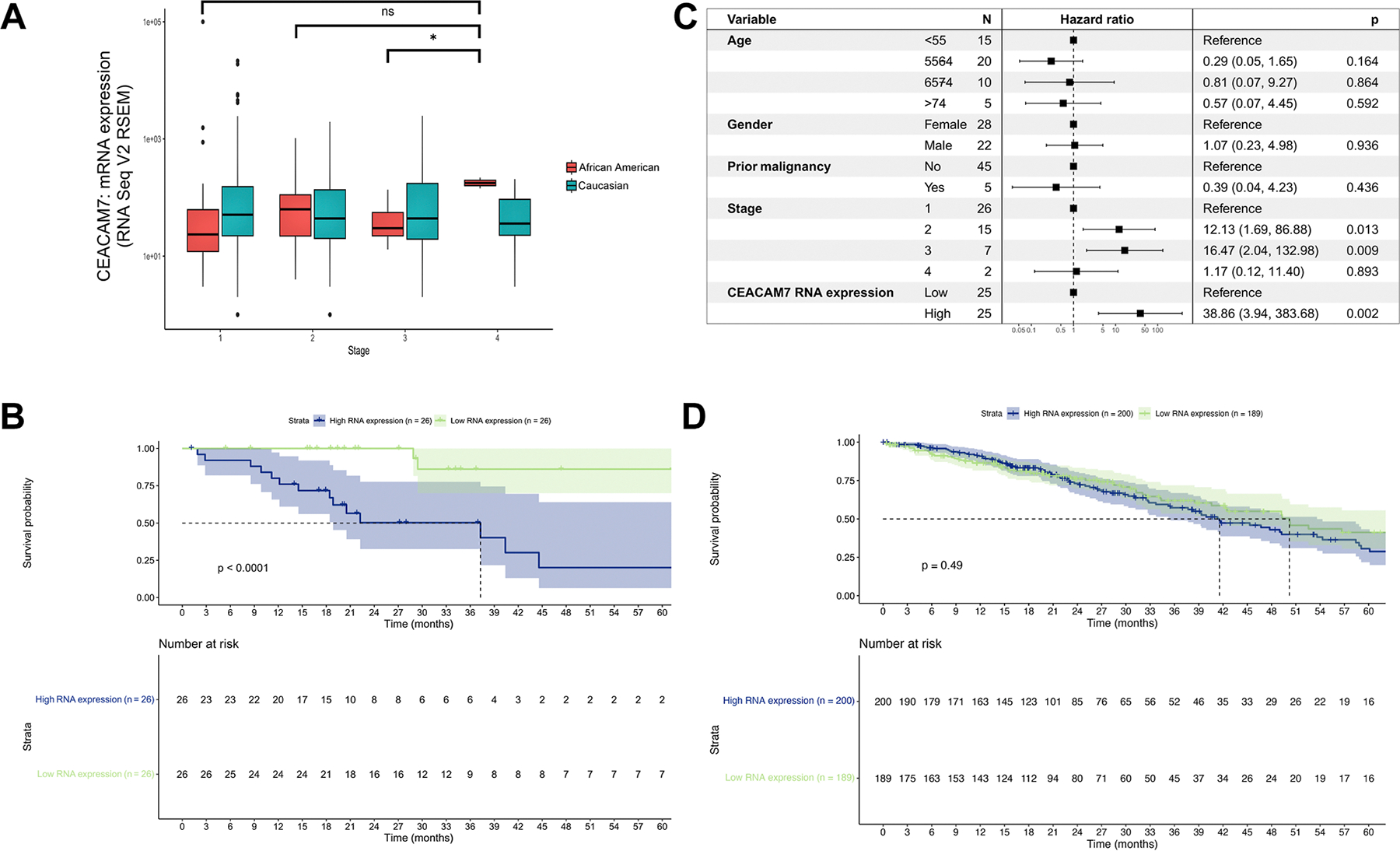
CEACAM7 expression is associated with later cancer stages, shorter survival, and increased mortality risk among African Americans with LUAD but not among Whites with LUAD. (*A*) Box plots for CEACAM7 expression by stage for African Americans and Whites with LUAD revealing that mRNA expression of CEACAM7 is higher in stage IV compared with stages I to III among African Americans with LUAD, but there are no differences in CEACAM7 RNA expression across stages for Whites with LUAD. (NS, nonsignificant; *p* < 0.10, *p < 0.05). (*B*) High CEACAM7 RNA expression had a significant shorter survival among African Americans with LUAD (*p* value < 0.0001) as found in the Kaplan-Meier curve with its corresponding log-rank test *p* value. (*C*) Forest plot revealing multivariate Cox proportional hazard analysis adjusting for age, sex, prior malignancy, and stage for African Americans with LUAD. High mRNA expression of CEACAM7 is associated with a significant increase in mortality risk (*p* value = 0.002). (*D*) CEACAM7 expression revealed no differences in survival for Whites patients with LUAD. LUAD, lung adenocarcinoma; LUSC, lung squamous cell carcinoma.

Next, we looked for survival differences for AA with LUAD and LUSC among the six up-regulated genes in AA, and only CEACAM7 (up-regulated in LUAD) demonstrated significant survival differences for AA with LUAD (Fig. 3). We further studied 52 AA with LUAD, comparing a group with high (n = 26) versus low (n = 26) CEACAM7 expression (Table 1). The median age at diagnosis was 60.5 (56–68.25) years for the high CEACAM7 group and 59.5 (51.5–68) years for the low CEACAM7 group. Both groups were homogeneous for baseline characteristics, without statistically significant differences for all variables. However, among AA with LUAD who had high CEACAM7 RNA expression, there was a trend toward having a significantly higher proportion of females (69%). In addition, mRNA expression of CEACAM7 at stage IV was the highest among AA with LUAD and was significantly higher than expression at stages I and III (Fig. 3A). However, there were no differences in CEACAM7 RNA expression across stages for Whites with LUAD.

### Survival Analysis CEACAM7 RNA Expression in African Americans

The two-tailed log-rank tests revealed that high expression of CEACAM7 in AA with LUAD had significantly shorter survival times when compared with low CECAM7 expression (p value < 0.0001) (Fig. 3B). Median survival was 37.29 months for the high CEACAM7 expression group and was not reached for the low RNA expression group after 5 years of follow-up. The 1-, 2-, and 5-year survival rates for the high CEACAM7 mRNA expression in AA with LUAD were 80%, 50%, and 20%, respectively, whereas the survival rates at the same time points for the low CEACAM7 mRNA expression group were 100%, 100%, and 86%, respectively.

Multivariate Cox proportional hazard model, adjusted for age, sex, prior malignancy, and stage, revealed that among AA with LUAD, high mRNA expression of CEACAM7 was significantly associated with an increased mortality risk, with HR of 38.86 (95% CI: 3.94–383.68, *p* = 0.002) (Fig. 3C).

In Whites with LUAD, there were no significant differences in overall survival from the log-rank test (*p* value = 0.49) when comparing high and low CEACAM7 mRNA expression (Fig. 3D). The median survival was 41.56 and 49.32 months, respectively. The 1-, 2-, and 5-year survival rates were 90%, 73%, and 30%, respectively, for the high expression group and 87%, 76%, and 41%, respectively, for the low expression group.

In AA with LUSC, there were no statistically significant differences in survival when comparing high versus low CEACAM7 mRNA expression (*p* = 0.52) (Supplementary Fig. 1A) and no significant association with mortality risk was found as well for AA with LUSC, with HR of 0.78 (95% CI: 0.17–3.68, *p* = 0.8) (Supplementary Fig. 1B).

When looking into the whole cohort of patients, including both Whites and AA, significantly shorter survival duration times were found for high CEACAM7 mRNA expression in LUAD (*p* = 0.038) (Supplementary Fig. 2A), but no significant survival duration differences were found when comparing high versus low CEACAM7 mRNA expression in LUSC (*p* = 0.85) (Supplementary Fig. 2B).

### CEACAM7 Differential Expression Analysis

**Figure 4.**
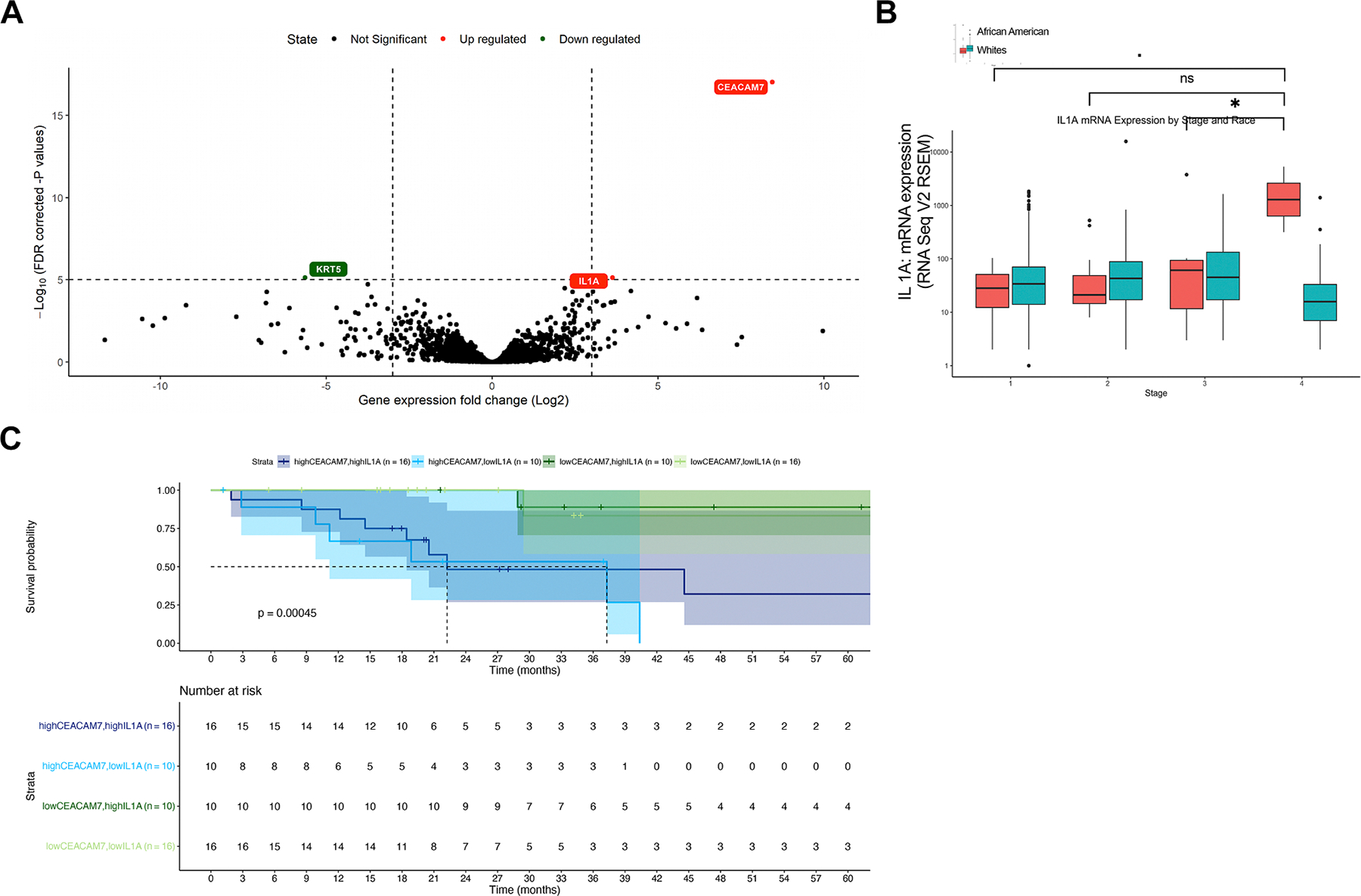
IL1A is overexpressed on patients with high CEACAM7 expression, and it is associated with late lung cancer stages and overall survival duration. (*A*) Volcano plot revealing up- and down-regulated genes when CEACAM7 is highly expressed for African Americans with LUAD (FDR cutoff = 10^−5^, log2FC = 3). (*B*) mRNA IL1A expression by AJCC pathologic stage on African Americans and Whites with LUAD (NS, nonsignificant; *p* < 0.10, *p < 0.05). (*C*) Kaplan-Meier survival curves comparing high CEACAM7 and high IL1A expression, high CEACAM7 and low IL1A, low CEACAM7 and high IL1A, and low CEACAM7 and low IL1A expression for African Americans with LUAD. AJCC, American Joint Committee on Cancer; FDR, false discovery rate; LUAD, lung adenocarcinoma; LUSC, lung squamous cell carcinoma.

Differential expression analysis of CEACAM7 mRNA expression, high versus low, identified one additional up-regulated gene, IL1A, and one down-regulated gene, KRT5, among AA with LUAD (FDR cutoff = 10−^5^, log_2_FC = 3) (Fig. 4A). When analyzing the mRNA expression of IL1A by lung cancer stage, we saw a similar picture to that of CEACAM7 expression; the mRNA expression of IL1A was higher in stage IV compared with stages I and III among AA with LUAD (Fig. 4B). However, there were no differences in IL1A RNA expression across stages for Whites with LUAD.

Then, we split AA with LUAD into four groups according to the mRNA expression level of both CEACAM7 and IL1A. Overall survival was significant (*p* value = 0.00045) among the four groups (Fig. 4C). Shorter overall survival was identified in two groups with high CEACAM7 expression in AA with LUAD. Median survival was 22.24 months for the high CEACAM7/high IL1A expression group, 37.29 months for the high CEACAM7/low IL1A expression group, and was not reached for either of the two groups with low CEACAM7 RNA expression (both high and low IL1A RNA expression).

### CEACAM7 Normal Tissue RNA Expression

To study expression of CEACAM7 in normal tissue, we used GTEx data across 53 normal tissue sites. The data revealed that the expression in normal lung tissue was low (0.08655 median transcripts per million [TPM]) along with other tissues, such as the stomach (0.07571 TPM) and whole blood (0.0326 TPM). The tissue with the highest CEACAM7 expression was the transverse colon (93.82 TPM) followed by vagina (30.61 TPM) and esophagus (30.30 TPM), whereas it was undetectable in the artery, brain, cultured fibroblast, heart, kidney, muscle, and nerve (Supplementary Fig. 3). The low normal expression of CEACAM7 in both the lung and whole blood makes it a good candidate for potential use in liquid biopsies.

### Correlation of CEACAM7 mRNA Expression and DNA Methylation

**Figure 5.**
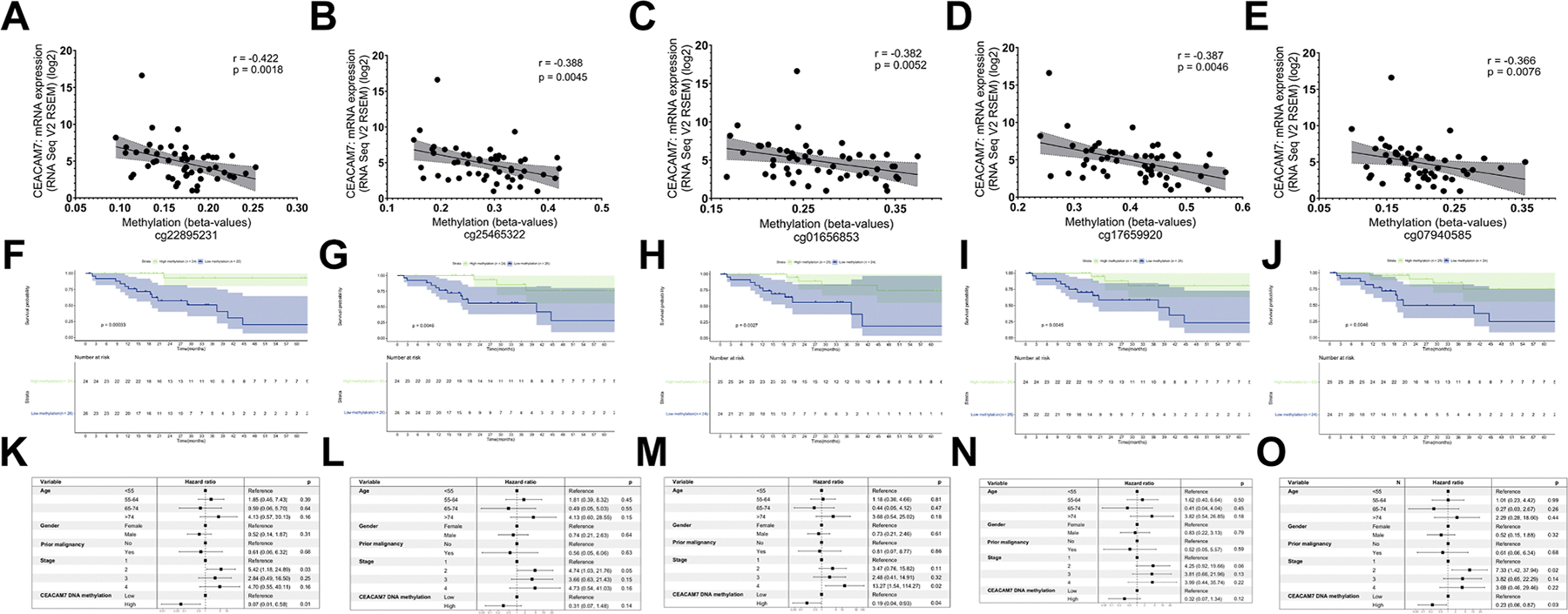
DNA methylation on CpG probes cg22895231, cg25465322, cg01656853, cg17659920, and cg07940585 is negatively correlated with CEACAM7 mRNA expression, directly associated with survival duration and inversely associated with mortality risk, respectively. (*A*–*E*) Scatter plots with Spearman’s correlation coefficients revealing the negative correlation between CEACAM7 mRNA expression and DNA methylation of (*A*) cg22895231, (*B*) cg25465322, (*C*) g01656853, (*D*) cg17659920, and (*E*) cg07940585. (*F*–*J*) Kaplan-Meier survival curves comparing high and low DNA methylation along with associated *p* values and numbers at risk tables for African Americans with LUAD for the following CpG probes: (*F*) cg22895231, (*G*) cg25465322, (*H*) cg01656853, (*I*) cg17659920, and (*J*) cg07940585. (*K*–*O*) Forest plots based of multivariate Cox proportional hazard analysis adjusting for age, sex, prior malignancy, and stage for African Americans with LUAD. Squares represent the hazard ratios and bars represent the 95% confidence intervals. Low DNA methylation is associated with worse survival for (*K*) cg22895231, (*L*) cg25465322, (*M*) cg01656853, (*N*) cg17659920, and (*O*) cg07940585. LUAD, lung adenocarcinoma; LUSC, lung squamous cell carcinoma.

Chromatin structure and DNA folding have been found to have a role in the regulation of distant gene expression by bringing distant genes into proximity and facilitating interactions between regulatory elements.^28–30^ CEACA7 is located on chromosome 19. Therefore, we assessed the correlation of the mRNA expression of CEACAM7 in LUAD with the DNA methylation of the CpG probes on entire chromosome 19 in AA. We identified five CpG probes (cg22895231, cg25465322, cg01656853, cg17659920, and cg07940585) that were statistically significantly negatively correlated with CEACAM7 RNA expression (Spearman’s r < −0.35, *p* value ≤ 0.01) (Fig. 5A–E). These probes were also significantly associated with survival according to the log-rank test (Fig. 5F–J) and univariate Cox proportional hazard models (Fig. 5K–O). In summary, we hypothesize that decreased mRNA expression of CEACAM7 could be regulated by increased DNA methylation of proximal CpG sites.

### Survival Analysis of DNA Methylation

Next, we studied the association between DNA methylation of the five *CEACAM7*-correlated probes with overall survival in AA with LUAD. Two-tailed log-rank tests revealed that high DNA methylation had significantly longer survival times for all probes (*p* value < 0.005). When DNA methylation was high, median survival was not reached after 5 years of follow-up for all five CpG probes. When DNA methylation was low, the median survival times for cg22895231, cg25465322, cg01656853, cg17659920, and cg07940585 were 37.29 months, 40.38 months, 37.29 months, 37.29 months, and 20.53 months, respectively. The 1-, 2-, and 5- year survival rates for high and low methylation of cg22895231, cg25465322, cg01656853, cg17659920, and cg07940585 were 100% and 80%, 93% and 57%, and 93% and 20% (Fig. 5F); 100% and 80%, 93% and 56%, and 76% and 28% (Fig. 5G); 100% and 78%, 89% and 56%, and 74% and 19% (Fig. 5H); 100% and 79%, 88% and 59%, and 81% and 23% (Fig. 5I); 96% and 82%, 90% and 50%, and 75% and 25% (Fig. 5J), respectively. Multivariate Cox proportional hazard analysis adjusting for age, sex, prior malignancy, and stage for AA with LUAD revealed significance in 60% of the probes: cg22895231 (HR: 0.07, 95% CI: 0.01–0.58, *p* = 0.01) (Fig. 5K), cg01656853 (HR: 0.19, 95% CI: 0.04–0.93, *p* = 0.04) (Fig. 5L), and cg07940585 (HR: 0.23, 95% CI: 0.06–0.87, *p* = 0.03) (Fig. 5M). No significance was found for cg25465322 (HR: 0.31, 95% CI: 0.07–1.48, *p* = 0.14) (Fig. 5N) and cg17659920 (HR: 0.32, 95% CI: 0.07–1.34, *p* = 0.12) (Fig. 5O).

### Pan-Cancer Differential Expression and Survival Analysis

We extended our analysis of CEACAM7 expression to all 32 TCGA cancer types to check whether its expression was significantly different between AA and Whites. The only cancer type where CEACAM7 expression was significantly higher in AA was LUAD.

In addition, we performed overall survival analysis using the two-tailed log-rank test on 15 cancer types that have a sufficient number of AA and Whites to test the association of the mRNA expression of CEACAM7 with overall survival (BLCA, BRCA, CESC, CHOL, COAD, ESCA, HNSC, KICH, PAAD, PRAD, READ, STAD, TGCT, UCEC, and UCS). That analysis demonstrated a significant association of CEACAM7 expression with overall survival (*p* value < 0.05) in BRCA (Supplementary Fig. 4) and READ (Supplementary Fig. 5).

In BRCA, high expression of CEACAM7 was associated with shorter overall survival in Whites (Supplementary Fig. 4C). In addition, the univariate Cox proportional hazard model indicated an increased mortality risk in Whites with BRCA (HR: 1.5, *p* = 0.048) (Supplementary Fig. 4F). However, high expression of CEACAM7 in READ demonstrated longer overall survival in all patients and Whites (Supplementary Fig. 5A and C). High expression of CEACAM7 in all patients with READ was also associated with a decreased mortality risk as determined by the univariate Cox proportional hazard model (HR: 0.3, *p* = 0.04) (Supplementary Fig. 5D).

## Discussion

This comprehensive genomic study reveals significant differences in lung cancer RNA expression between AA and Whites. Notably, CEACAM7 was the only gene overexpressed in AA with LUAD, with its mRNA expression significantly linked to lung cancer stage, overall survival duration, and mortality risk. We found that DNA methylation on five CpG probes (cg22895231, cg25465322, cg01656853, cg17659920, and cg07940585) was negatively correlated with CEACAM7 mRNA expression. These methylation patterns were directly associated with survival duration and inversely associated with mortality risk. In addition, high CEACAM7 expression in AA with LUAD was associated with elevated IL1A expression. The co-expression of CEACAM7 and IL1A was linked to the shortest overall survival duration for AA with LUAD. In a pan-cancer analysis of 32 cancer types in TCGA, CEACAM7 was not differentially expressed between AA and Whites, except in LUAD. CEACAM7 expression was also associated with survival in BRCA and READ.

### Mechanism of Action

CEACAM7 is a GPI-anchored protein that is part of the carcinoembryonic antigen-related cell adhesion molecule (CEACAM) family. Proteins from the CEACAM family have been found to regulate angiogenesis, tumor invasion, and metastasis in cancer.^31–33^ In addition, CEACAM family mutations have been found to be associated with inherited cancer risk for breast and colorectal cancer, respectively.^34,35^ In particular, CEACAM7 has the potential to be a prognostic marker in various cancers and a target for CAR T-cell therapy to treat pancreatic ductal adenocarcinoma.^36–38^ As a surface glycoprotein, CEACAM7 may play a key role in extracellular matrix signaling in LUAD, BRCA, and READ, thereby leading to associations with survival in these cancer types. Further insight into CEACAM7 and its molecular pathways may help researchers better understand the exact mechanisms by which CEACAM7 affects LUAD prognosis. These pathways, along with CEACAM7 itself, may prove to be valuable targets in the treatment of LUAD, especially because it appears to disproportionately affect AA. However, the role of CEACAM7 in NSCLCs, particularly among patients with AA, has not been previously studied.

### CEACAM7 and IL1A

IL1A, or interleukin-1 alpha, is part of the interleukin-1 cytokine family. It has most notably been studied as part of the innate immune response, an important molecule in the inflammatory conditions of the tumor microenvironment.^39^ To date, no clear explanation exists explaining the observed relationship between CEACAM7 mRNA expression and IL1A mRNA expression.^40^ In our differential expression analysis, as found in volcano plots, IL1A expression was increased with elevated CEACAM7 mRNA expression (Fig. 4A). In addition, the CEACAM7-IL1A co–up-regulation also appears to be associated with overall shorter survival duration, as the high CEACAM7 and high IL1A expression group had shorter overall survival compared with the low CEACAM7 and low IL1A expression group (22.24 mo versus 37.29 mo) (Fig. 4C).

### Epigenetics

Epigenetic modulation of CEACAM7 also appears to play a role in CEACAM7 mRNA expression, overall survival, and mortality risk. Five DNA methylation sites (cg22895231, cg25465322, cg01656853, cg17659920, and cg07940585) were correlated with CEACAM7 expression. These methylation sites were also associated with overall survival in AA with LUAD (*p* value < 0.005), and when adjusting for covariates, age and cancer stage appeared to have the biggest associations with decreased DNA methylation (Fig. 5). Despite adjustment, low DNA methylation at these sites was often inversely correlated with overall survival risk. In addition to the observation that there were no significant differences in overall survival among Whites with high or low CEACAM7 mRNA expression (*p* value = 0.5), the same was not true for AA. The data imply that there are unobserved biological or environmental factors that affect CEACAM7-associated LUAD prognosis in AA. Because our analysis did not identify clear molecular pathways linking CEACAM7 to the LUAD tumor microenvironment aside from possible inflammation, there are—as many would expect—likely key environmental factors that correlate with CEACAM7 expression and/or LUAD prognosis. Such environmental factors include diet, lifestyle, and toxin exposure.^41^

### Limitations

As with any study, omitted variables can bias results. For example, disparities in socioeconomic conditions that lead to more toxin exposure and decreased access to care may be overrepresented in this cohort, resulting in upward bias or an overestimation of the ability of CEACAM7 to predict morbidity and mortality.^42^ Because the TCGA data set was clinically limited to variables such as age, sex, race, pack*year smoked, years smoked, prior malignancy, histology, and AJCC cancer stage, no adjustment could be made to control for potential confounders such as socioeconomic status.

Ostensibly, larger sample sizes are necessary to better inform any conclusion. As such, another limitation of this study was the disproportionately low percentage of samples from AA among different cancer types in the TCGA. The TCGA database had only 12% of AA making up the LUAD sample, with only 26 AA in the high-expression group and 26 AA in the low-expression group, thereby limiting the generalizability of potential findings.

### Clinical Impact and Future Directions

The clinical implications of a prognostic marker, such as CEACAM7, would help stratify patients into high- or low-risk groups. Future studies could benefit from intentionally enriching cohorts with higher percentages of AA patients with lung cancer to further explore population-specific genomic and clinical insights. With more information about a patient’s particular clinical course, clinicians can personalize their therapeutic approaches.^43^ Furthermore, the use of biomarkers can help predict tumor response to therapeutic approaches and prevent the improper treatment of patients with lung cancer, either excessive or insufficient. Similarly, clinical risk prediction tools could inform cohort selection for chemoprevention studies or as surrogate endpoints for prevention intervention investigations.^44^

Epigenetic biomarkers, in particular, have certain advantages compared with genetic biomarkers as they can incorporate environmental information, are often stable in various fluids, and can withstand types of tissue preparations.^45^ Furthermore, because normal lung tissue and whole blood demonstrate low CEACAM7 expression based on GTEx data, CEACAM7 may be a good candidate for possible use in liquid biopsies. Additional testing to determine clinical utility, such as positive predictive value, would be necessary.^44^

Finally, targeting CEACAM7 itself may be a therapeutic target in the treatment of LUAD. CEACAM7 CAR T-cells are already being developed and have demonstrated the ability to target antigen-expressing pancreatic ductal adenocarcinoma. Targeting LUAD with the same therapy would be a natural next step. In addition, one common barrier to CAR T-cell therapy is that tumor antigens are often also expressed in normal tissues, typically at low levels. However, CEACAM7, as aforementioned, has low to no detectable expression in normal tissues.^38,46^

In conclusion, CEACAM7 RNA expression and its associated DNA methylation are associated with overall survival duration and mortality risk among AA with LUAD, but not among Whites.

## Supplementary Material

MMC1

MMC2

figs13

figs4

figs1

figs12

figs9

figs8

figs6

figs3

figs7

figs11

figs5

figs2

figs14

figs15

figs10

figs16

Supplementary Data

Note: To access the supplementary material accompanying this article, visit the online version of the *Journal of Thoracic Oncology* at www.jto.org and at https://doi.org/10.1016/j.jtho.2025.08.015.

## Figures and Tables

**Table 1.** Baseline Characteristics of 52 African Americans With LUAD

Patient Characteristics	High CEACAM7 RNA (n = 26)	Low CEACAM7 RNA (n = 26)	*p* Value

Age (y) (IQR)	60.5 (56–68.25)	59.5 (51.5–38)	0.627
Gender			
Male (%)	8 (31)	15 (58)	0.093
Female (%)	18 (69)	11 (42)	
Pack*year (IQR)	20 (10.75–32.5)	30 (20–41.5)	0.108
Years smoked (IQR)	24 (13.75–32.25)	30 (30–38)	0.264
Prior malignancy (%)	3 (12)	2 (8)	1
Stage			
I (%)	10 (38)	16 (62)	0.170
II (%)	10 (38)	5 (19)	
III (%)	3 (12)	4 (15)	
IV (%)	2 (8)	0 (0)	

IQR, interquartile range; LUAD, lung adenocarcinoma.

## Data Availability

The data set supporting the conclusions of this article is available in the TCGA repository: https://cancergenome.nih.gov/. The RNA sequencing data used were obtained from the Genotype-Tissue Expression (GTEx) Project Portal dbGaP accession phs000424.v8.p2 GSE2361. The code used to conduct the analysis of this study is available at reasonable request.
