# Supplementary figures and images for: CEACAM7 Expression and DNA Methylation: Prognostic Biomarkers for Lung Adenocarcinoma in African Americans
Source: J Thorac Oncol. Author manuscript; Available in PMC 2026 Jul 22. (PMC13390726; doi:10.1016/j.jtho.2025.08.015)

A

## BRCA all

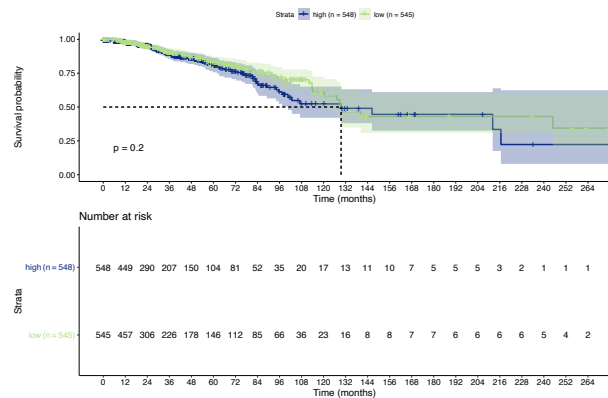

B

## BRCA African Americans

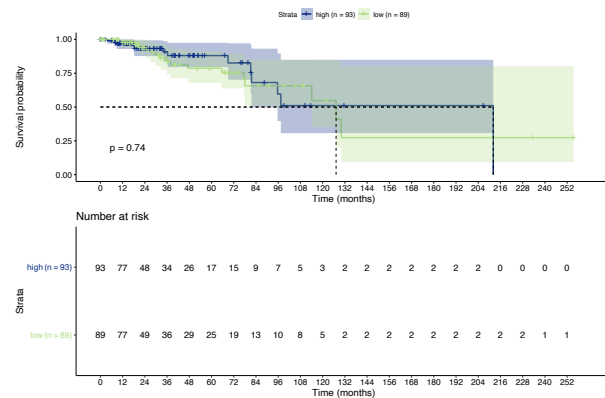

C

## BRCA Whites

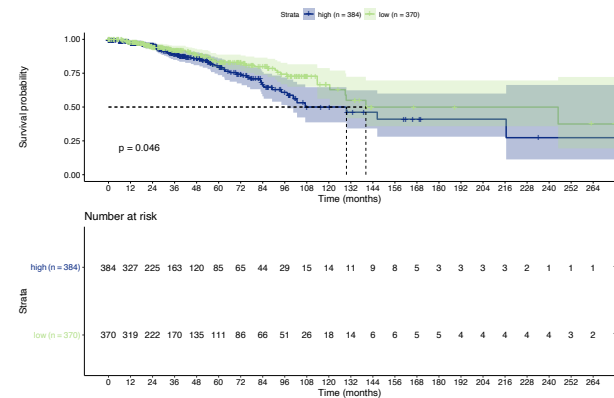

D

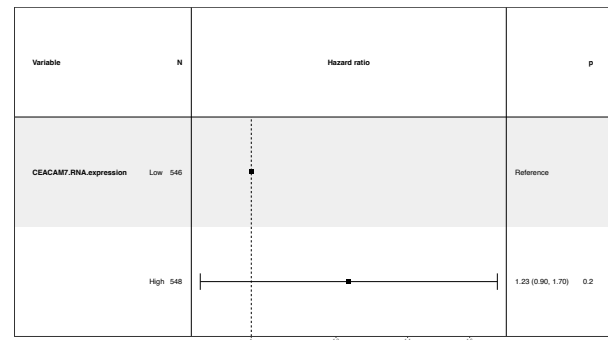

E

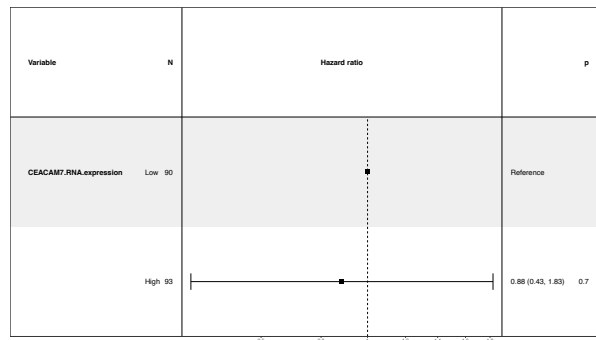

F

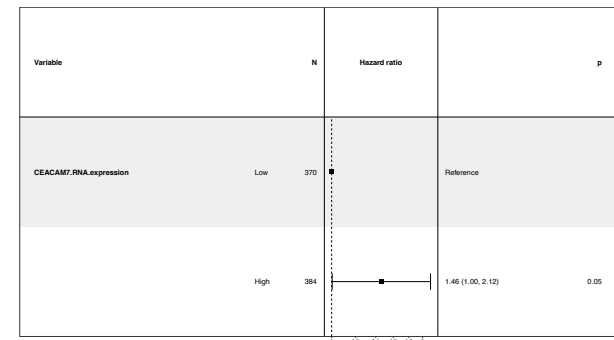

Supplement: MMC1 [file NIHMS2182458-supplement-MMC1.pdf]

# A

## READ all

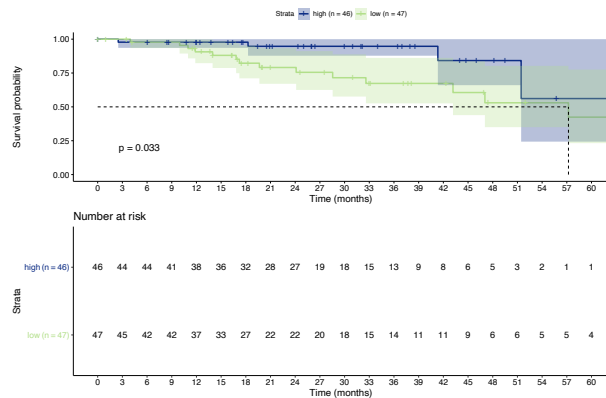

# B

## READ African americans

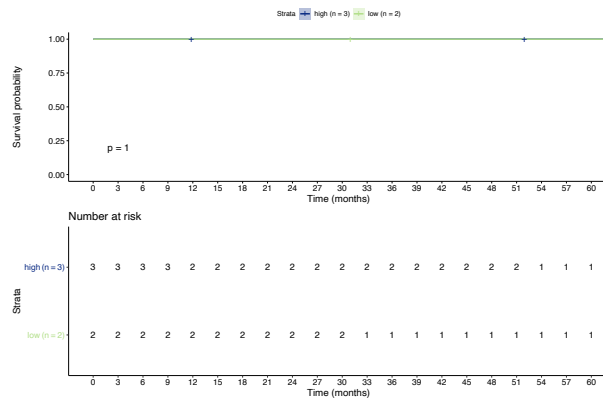

# C

## READ Whites

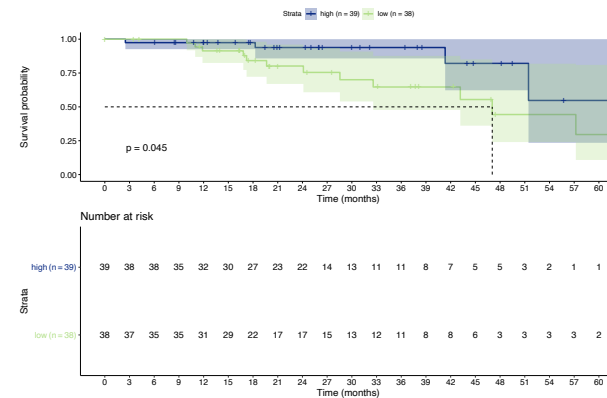

# D

## READ all

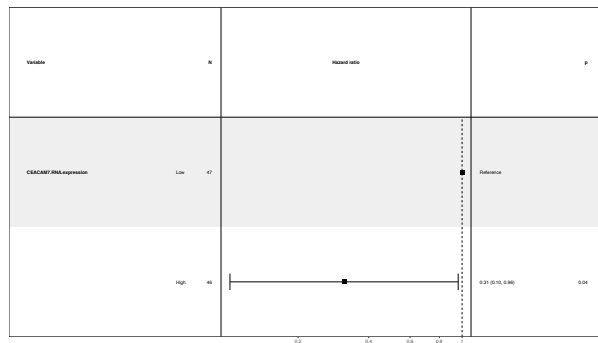

# E

## READ Whites

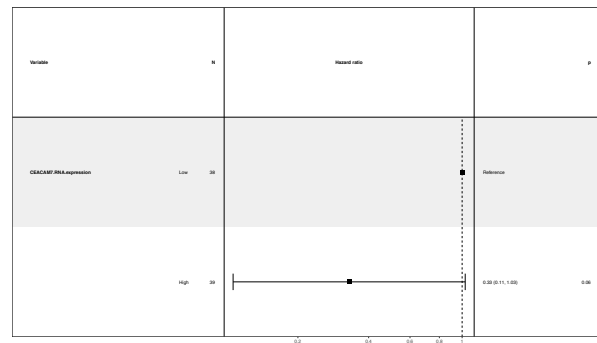

Supplement: MMC2 [file NIHMS2182458-supplement-MMC2.pdf]

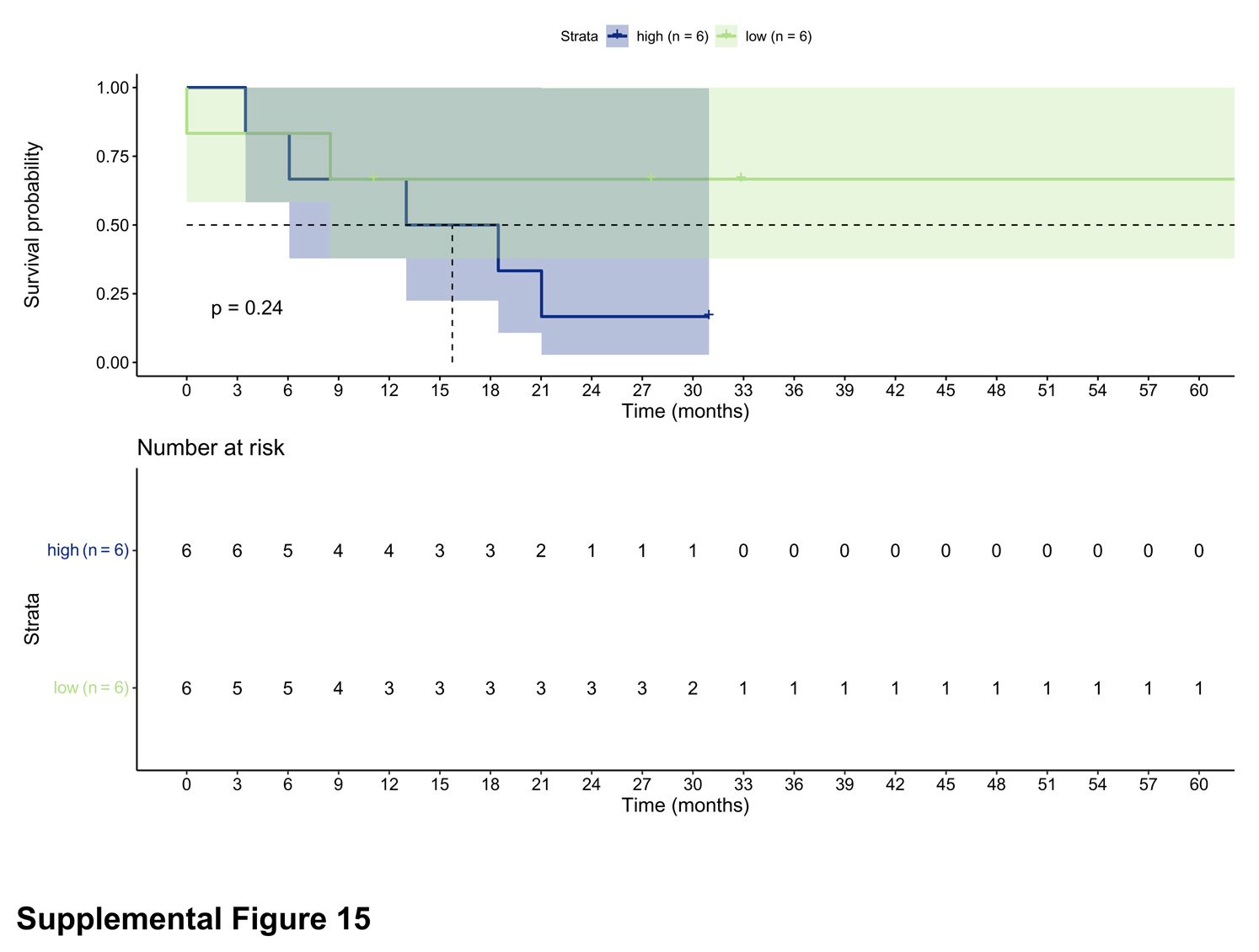

Supplement: figs13 [file NIHMS2182458-supplement-figs13.jpg]

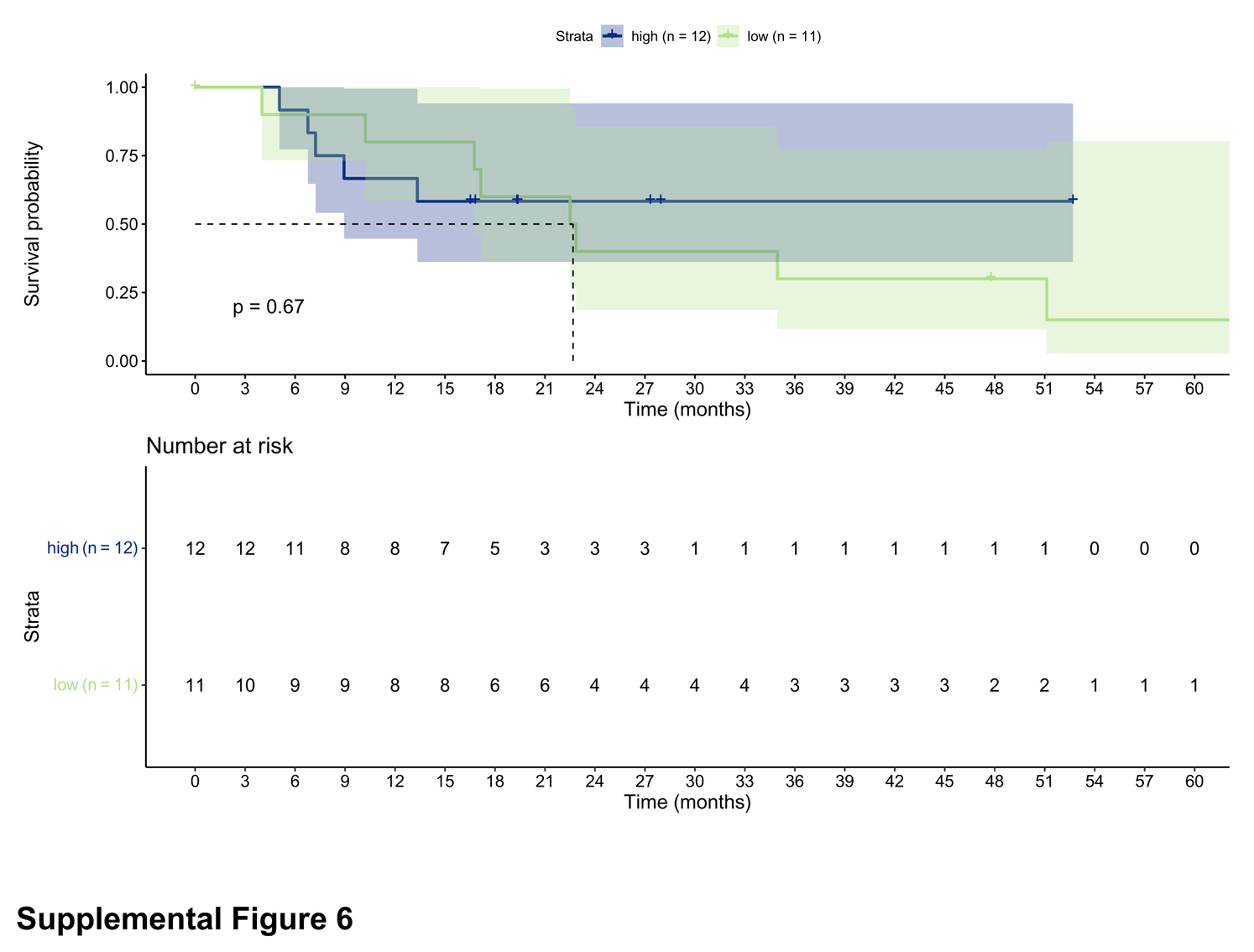

Supplement: figs4 [file NIHMS2182458-supplement-figs4.jpg]

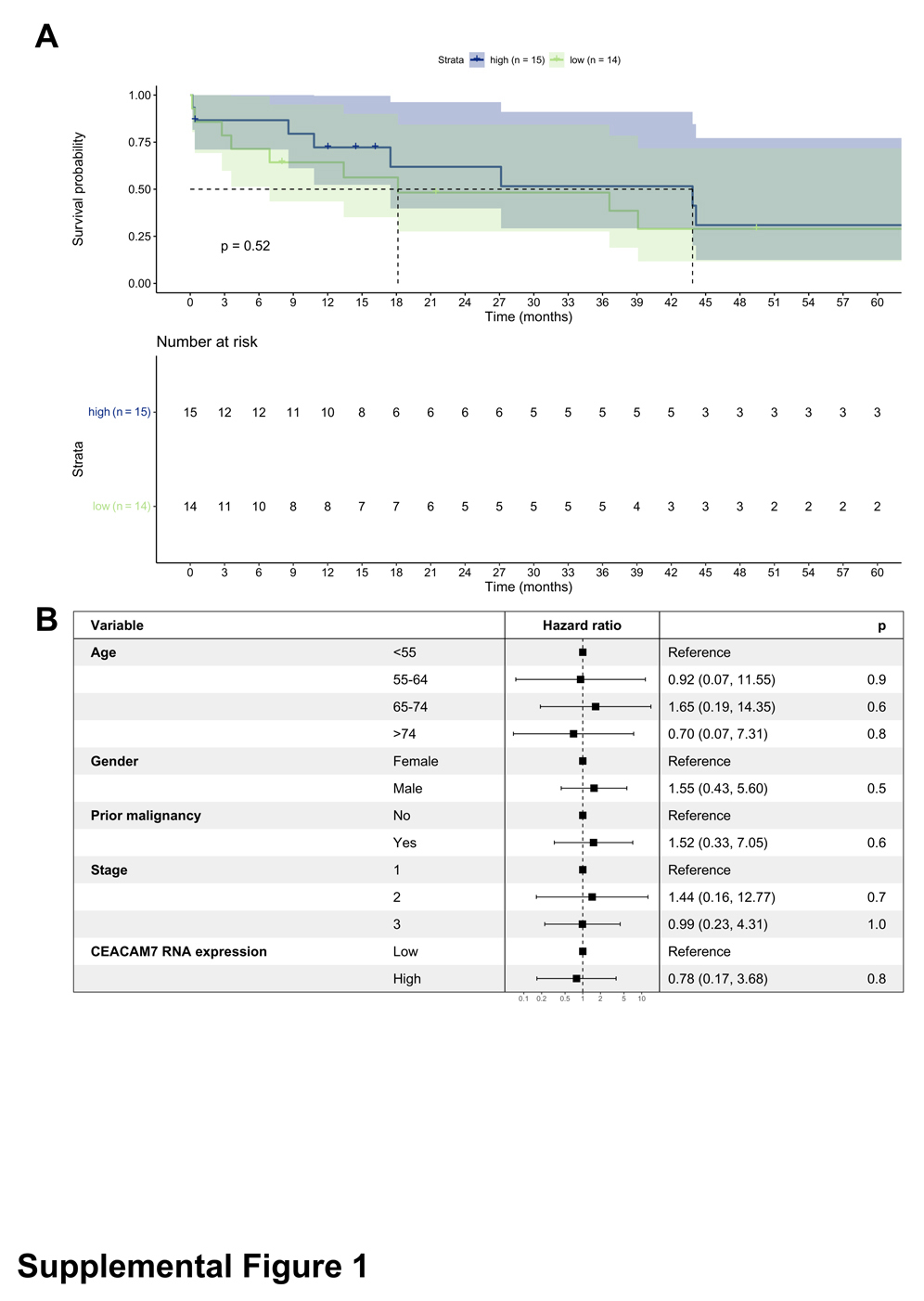

Supplement: figs1 [file NIHMS2182458-supplement-figs1.jpg]

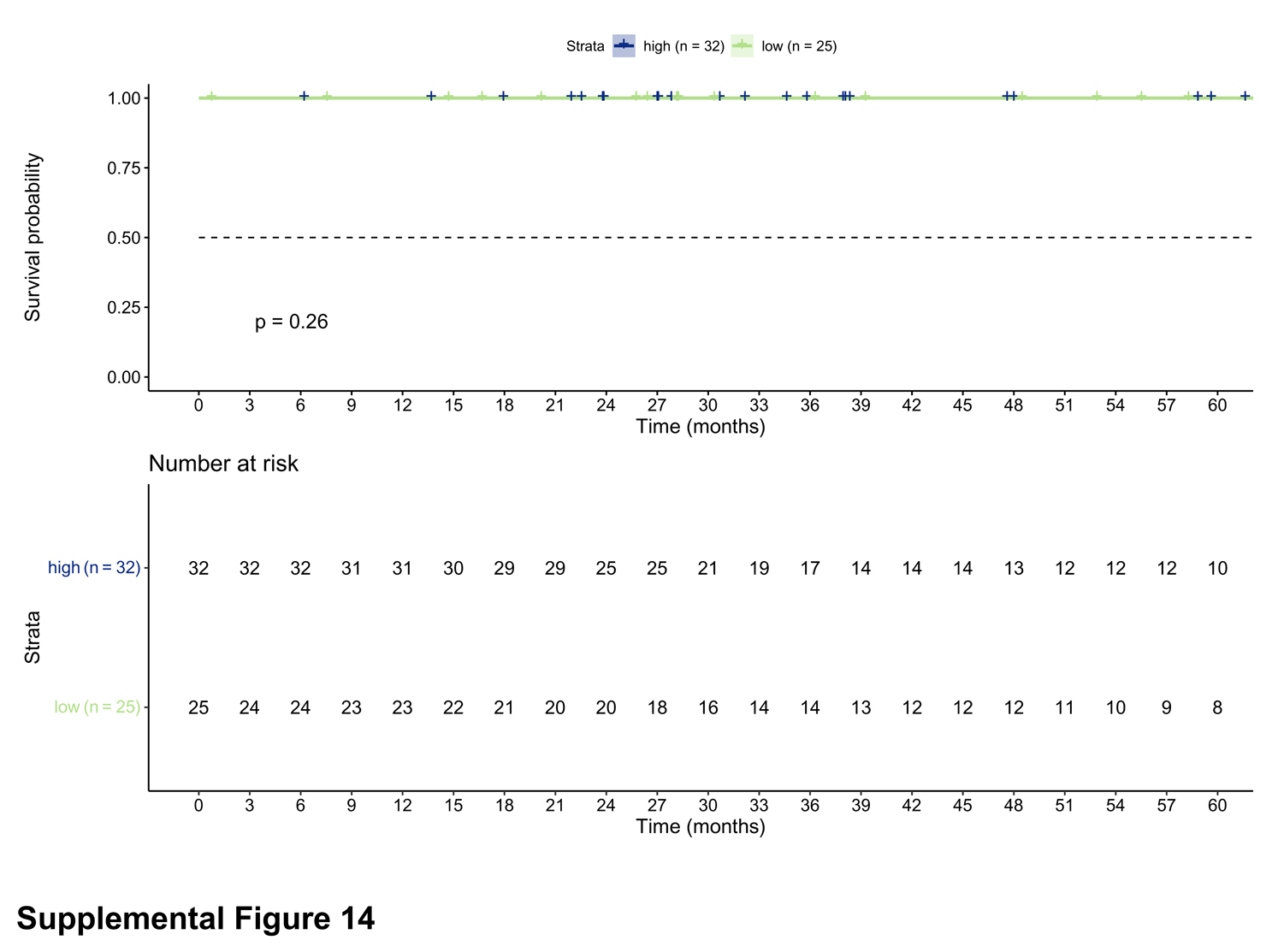

Supplement: figs12 [file NIHMS2182458-supplement-figs12.jpg]

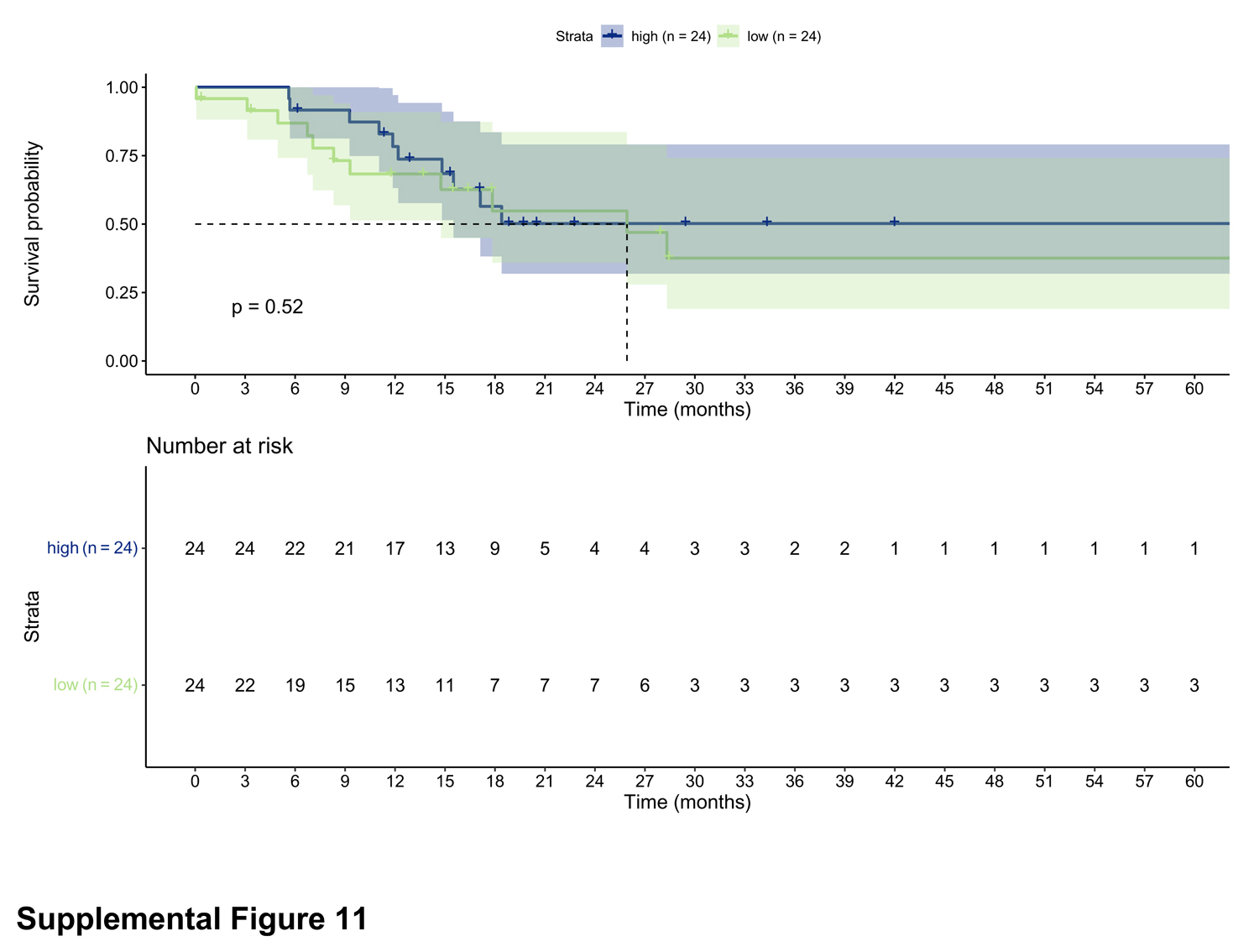

Supplement: figs9 [file NIHMS2182458-supplement-figs9.jpg]

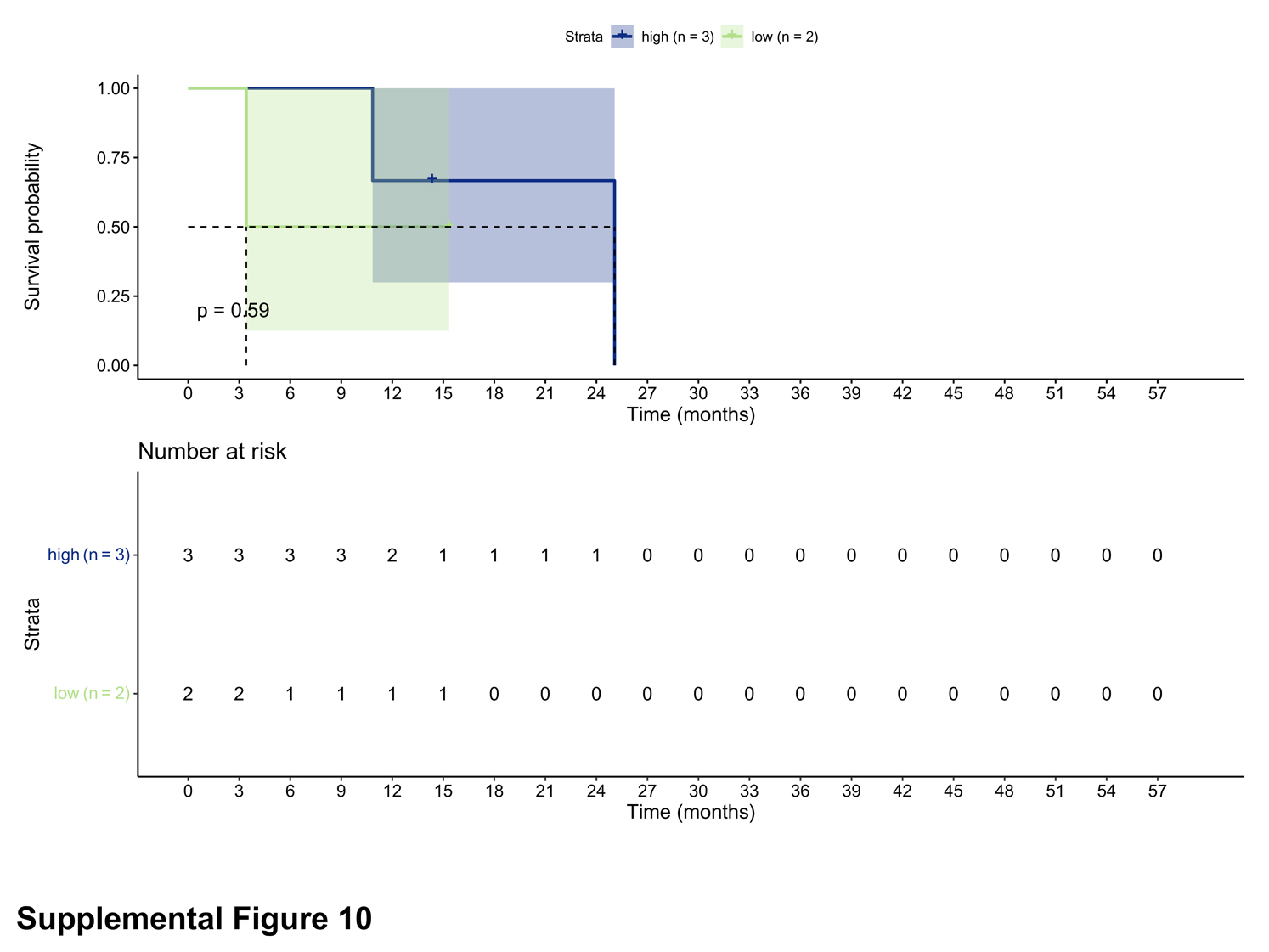

Supplement: figs8 [file NIHMS2182458-supplement-figs8.jpg]

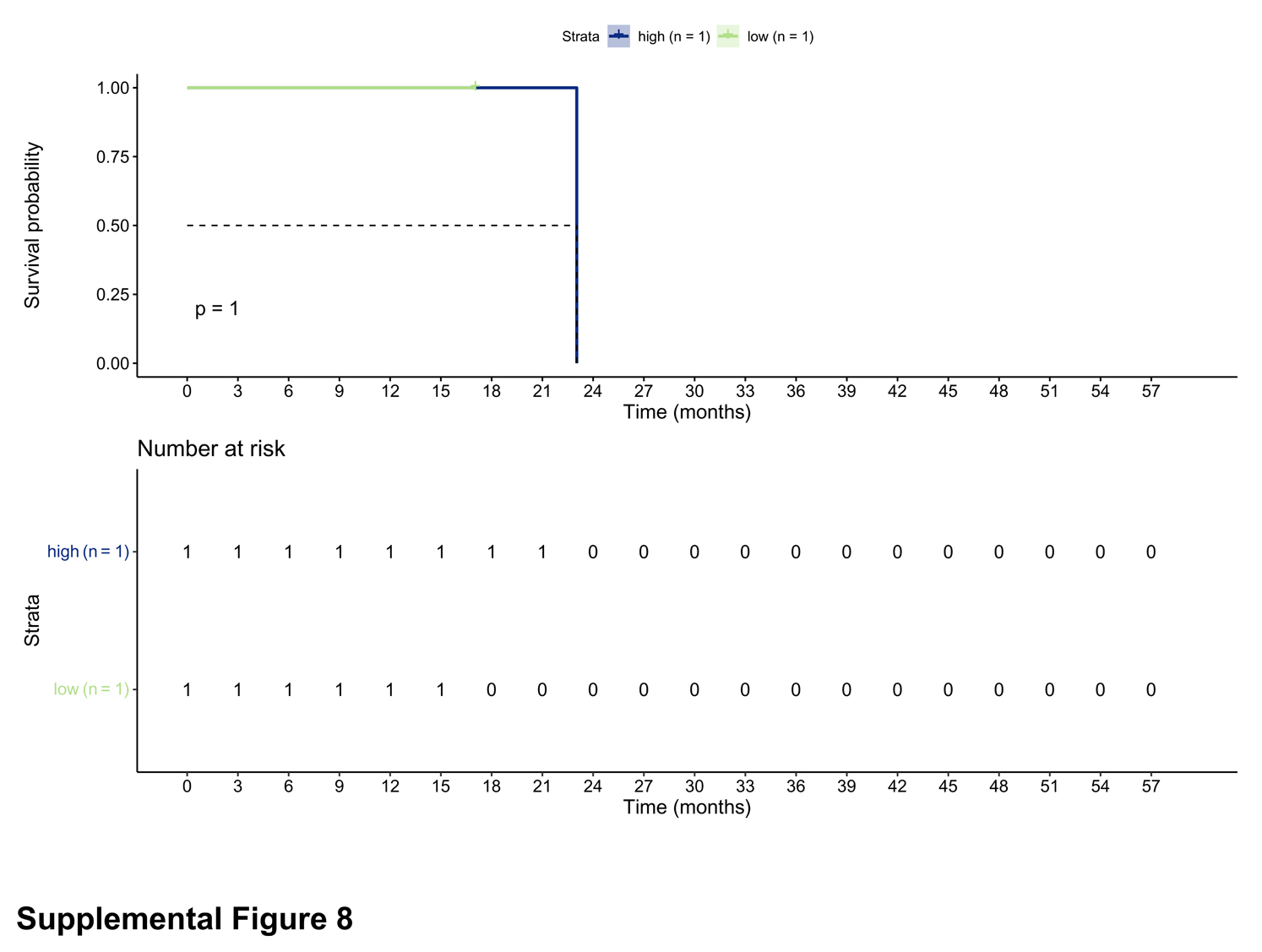

Supplement: figs6 [file NIHMS2182458-supplement-figs6.jpg]

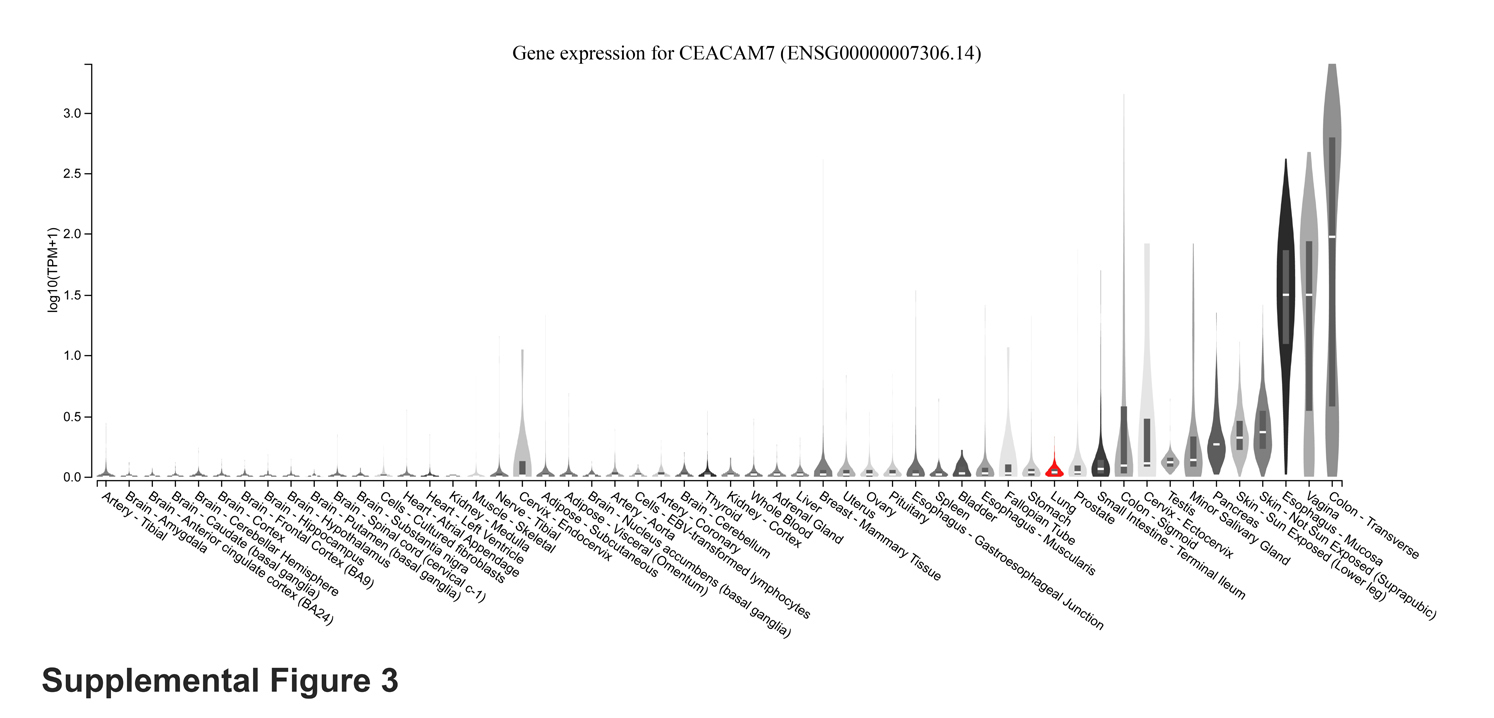

Supplement: figs3 [file NIHMS2182458-supplement-figs3.jpg]

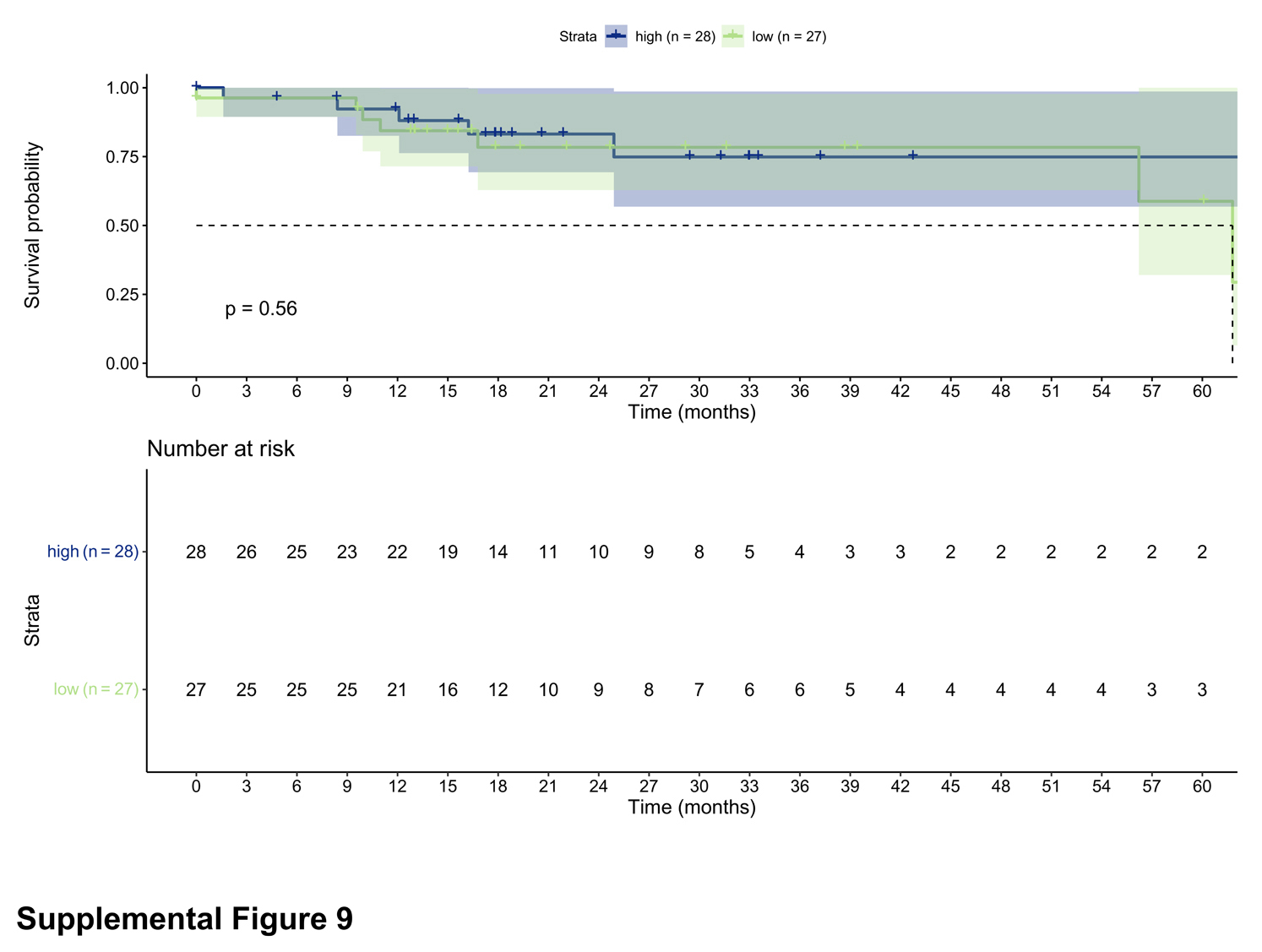

Supplement: figs7 [file NIHMS2182458-supplement-figs7.jpg]

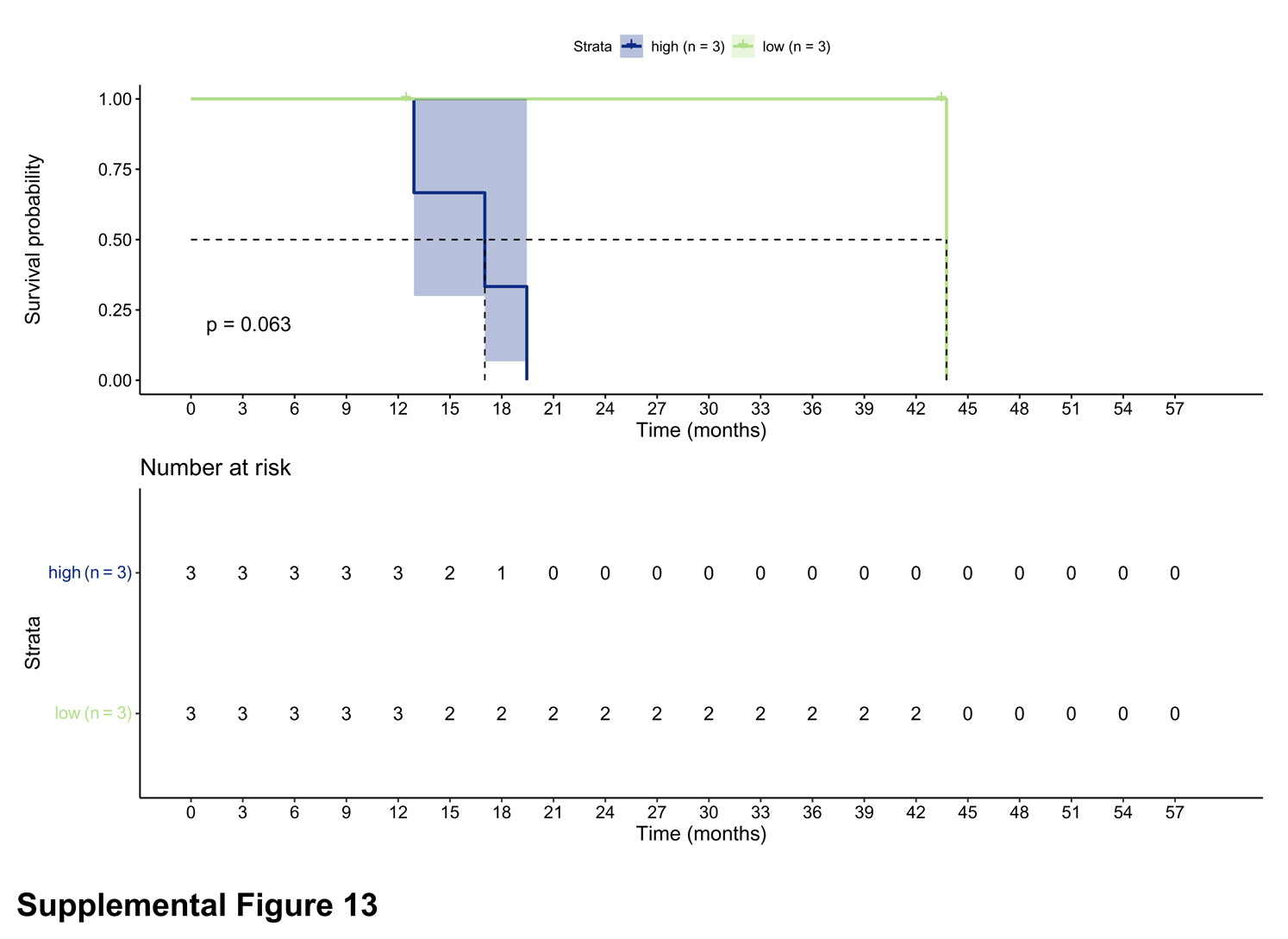

Supplement: figs11 [file NIHMS2182458-supplement-figs11.jpg]

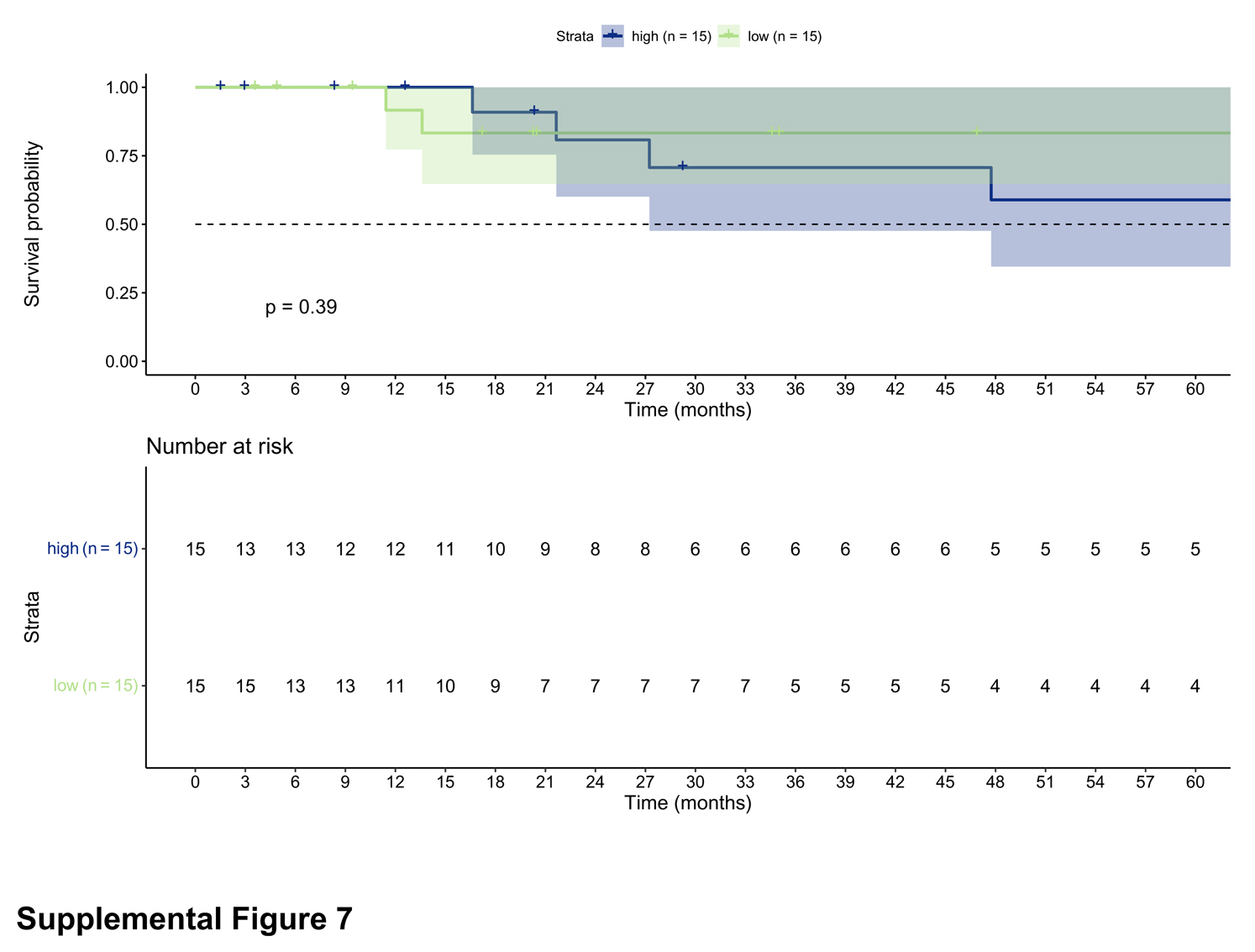

Supplement: figs5 [file NIHMS2182458-supplement-figs5.jpg]

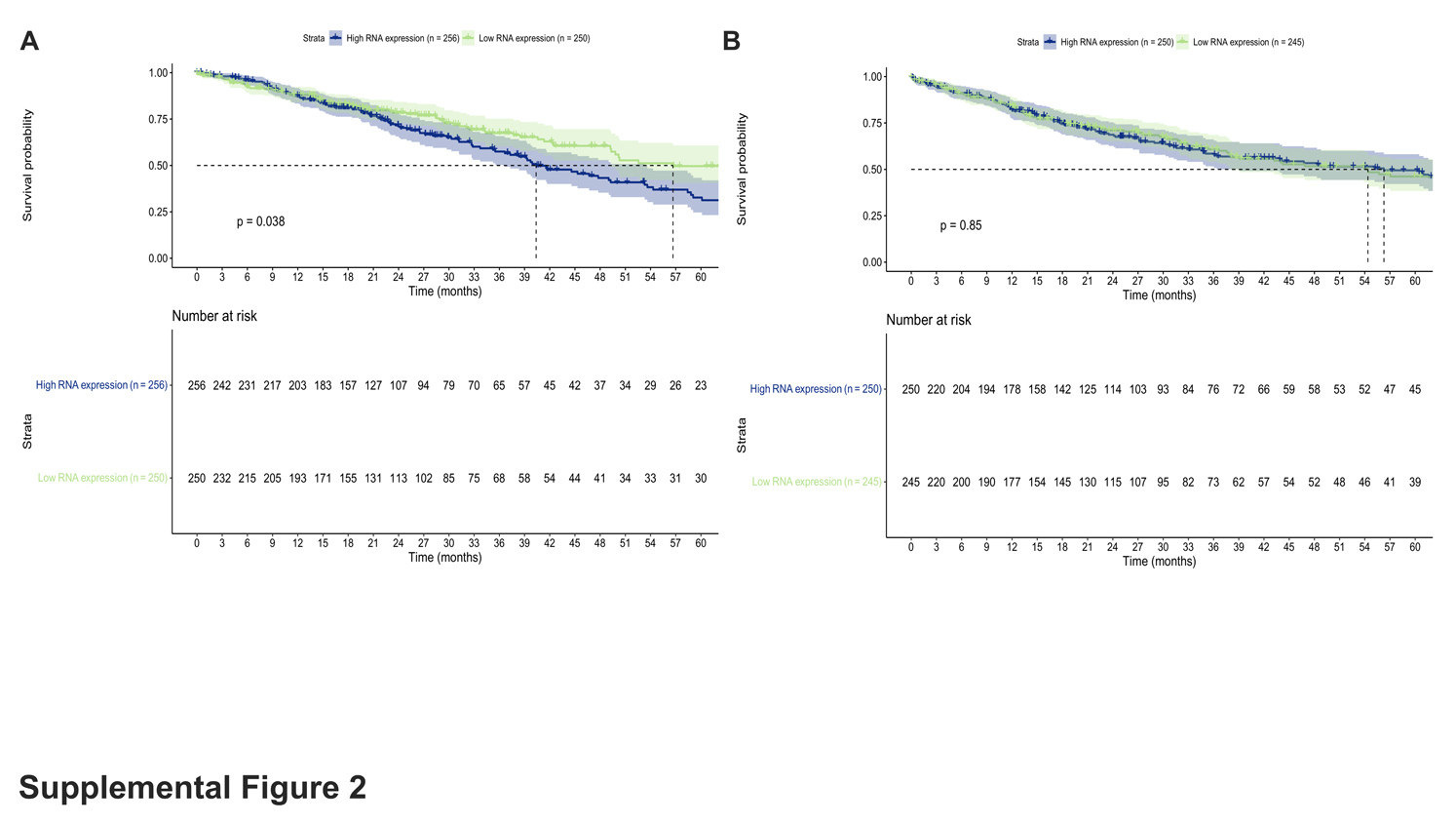

Supplement: figs2 [file NIHMS2182458-supplement-figs2.jpg]

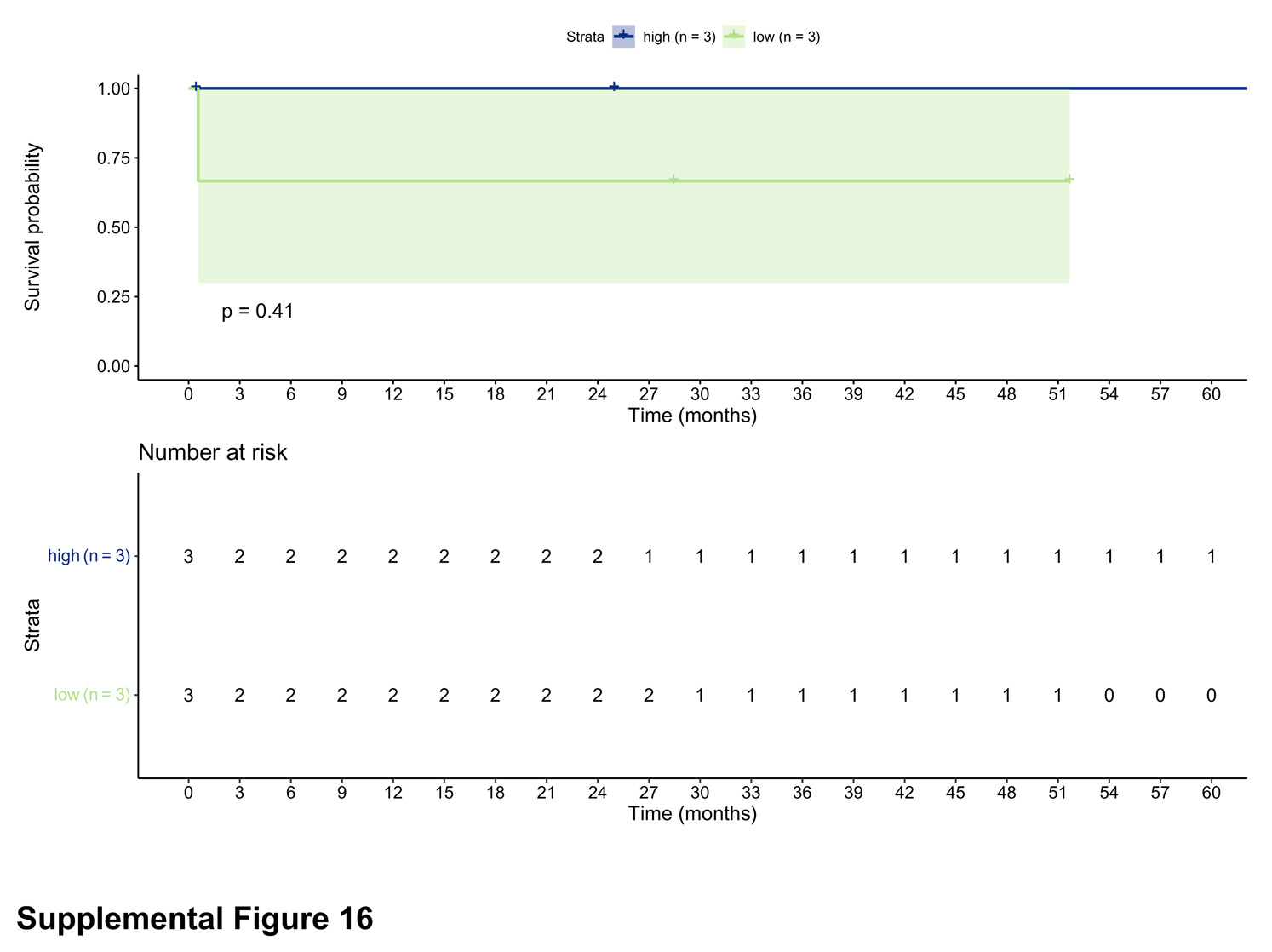

Supplement: figs14 [file NIHMS2182458-supplement-figs14.jpg]

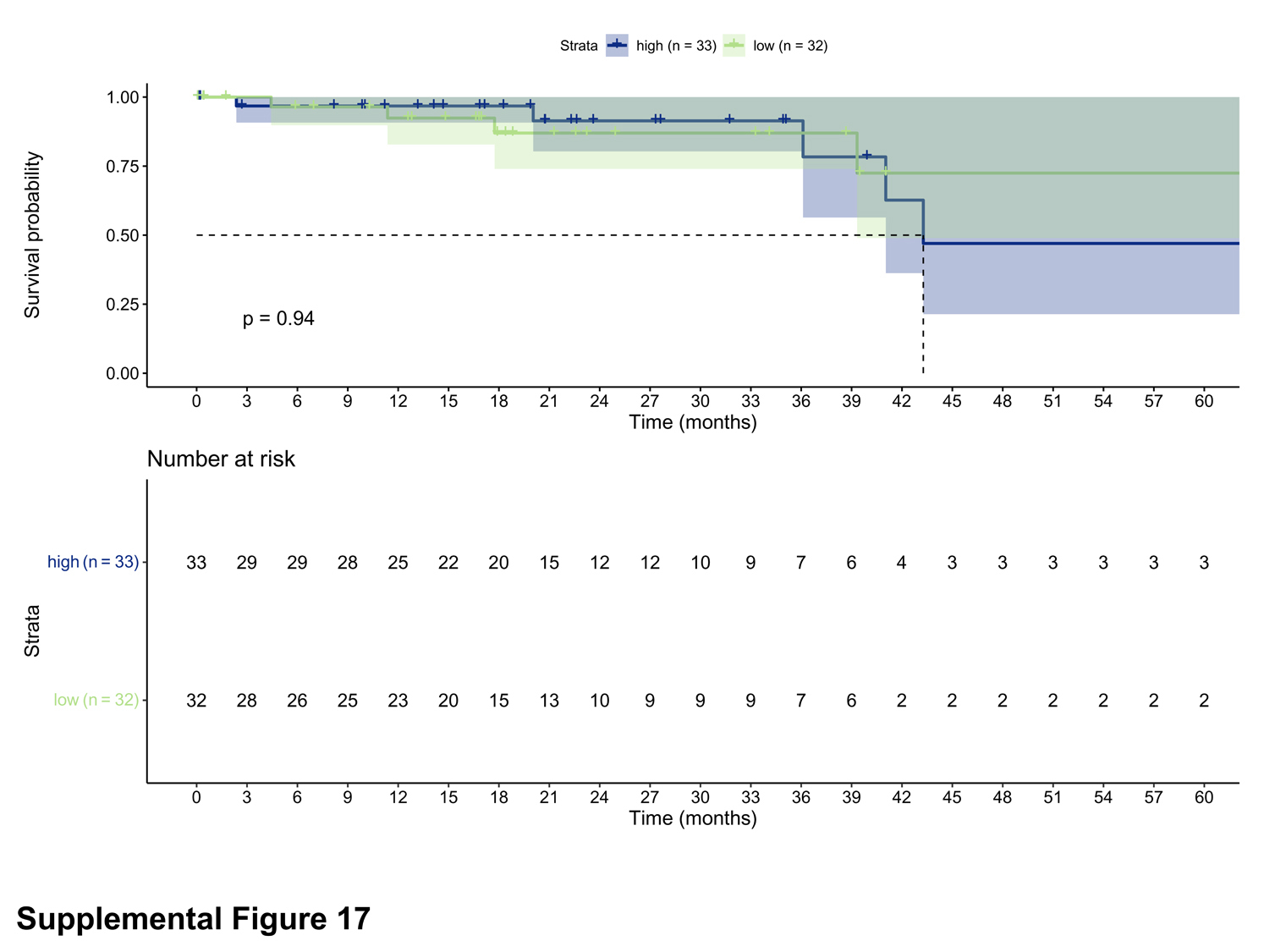

Supplement: figs15 [file NIHMS2182458-supplement-figs15.jpg]

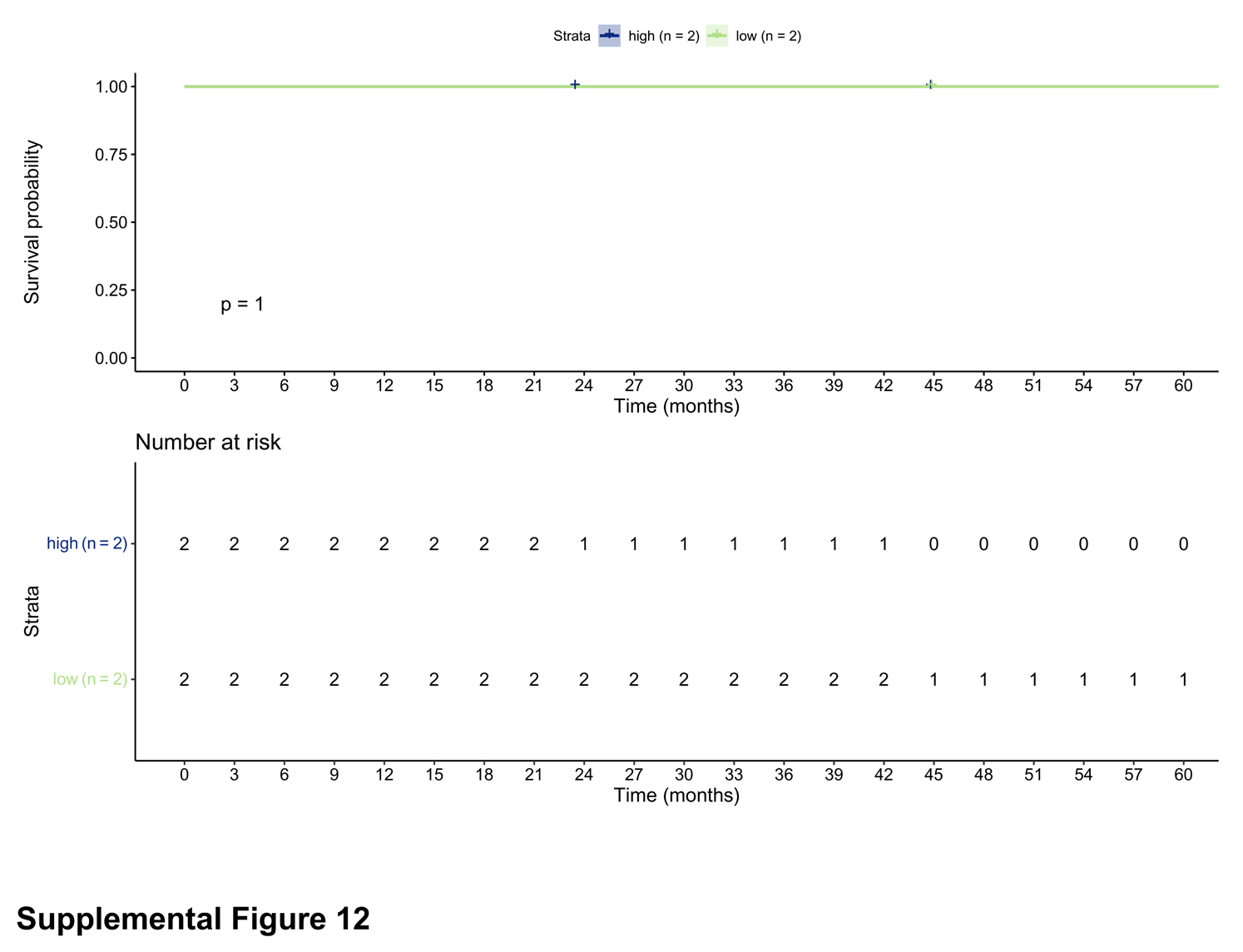

Supplement: figs10 [file NIHMS2182458-supplement-figs10.jpg]

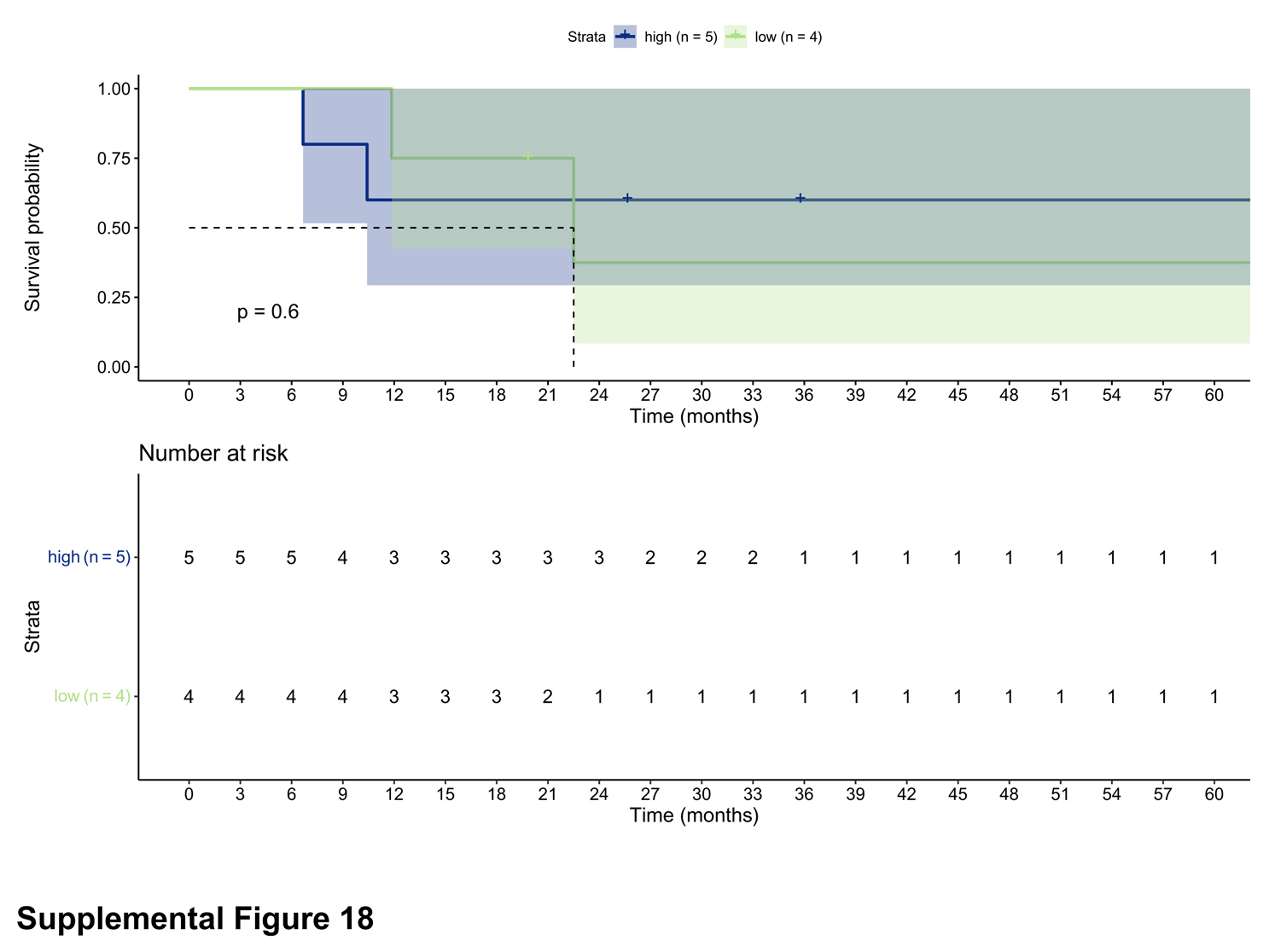

Supplement: figs16 [file NIHMS2182458-supplement-figs16.jpg]
